# Imaging with a twist: Three-dimensional insights of the chiral nematic phase of cellulose nanocrystals via SHG microscopy

**DOI:** 10.1126/sciadv.adp2384

**Published:** 2024-10-30

**Authors:** Thibaut Legat, Vladimir Grachev, Desmond Kabus, Minne Paul Lettinga, Koen Clays, Thierry Verbiest, Yovan de Coene, Wim Thielemans, Stijn Van Cleuvenbergen

**Affiliations:** ^1^ Molecular Imaging and Photonics, Department of Chemistry, KU Leuven, Campus Kulak Kortrijk, Etienne Sabbelaan 53, 8500 Kortrijk, Belgium.; ^2^ Sustainable Materials Lab, Department of Chemical Engineering, KU Leuven, Campus Kulak Kortrijk, Etienne Sabbelaan 53, 8500 Kortrijk, Belgium.; ^3^ Department of Mathematics, KU Leuven, Campus Kulak Kortrijk, Etienne Sabbelaan 53, 8500 Kortrijk, Belgium.; ^4^ Laboratory of Experimental Cardiology, Leiden University Medical Center (LUMC), Albinusdreef 2, 2333 ZA Leiden, Netherlands.; ^5^ Laboratory for Soft Matter and Biophysics, KU Leuven, Leuven, Belgium.; ^6^ Biological Information Processing IB-4, Forschungszentrum Jülich, 52425 Jülich, Germany.; ^7^ Molecular Imaging and Photonics, Department of Chemistry, KU Leuven, Celestijnenlaan 200D, 3001 Heverlee, Belgium.

## Abstract

Cellulose nanocrystals (CNCs) are bio-based nanoparticles that, under the right conditions, self-align into chiral nematic liquid crystals with a helical pitch. In this work, we exploit the inherent confocal effect of second-harmonic generation (SHG) microscopy to acquire highly resolved three-dimensional (3D) images of the chiral nematic phase of CNCs in a label-free manner. An in-depth analysis revealed a direct link between the observed variations in SHG intensity and the pitch. The highly contrasted 3D images provided unprecedented detail into liquid crystal’s native structure. Local alignment, morphology, as well as the presence of defects are readily revealed, and a provisional framework relating the SHG response to the orientational distribution of CNC nanorods within the liquid crystal structure is presented. This paper illustrates the numerous benefits of using SHG microscopy for visualizing CNC chiral nematic systems directly in the suspension-liquid phase and paves the road for using SHG microscopy to characterize other types of aligned CNC structures, in wet and dry states.

## Teaser

Second-harmonic generation imaging of self-aligned cellulose nanocrystals reveals natural deviations and intricate 3D structures.

## INTRODUCTION

Cellulose is a ubiquitous biomaterial made up of β−1,4-linked anhydro-d-glucose units and is the main constituent of higher order plants, several marine animals, fungi, and amoeba (*1*). It acts, for the most part, as the structural component of the cell walls of these organisms. Cellulose is a semicrystalline material with the elementary fibrils consisting of crystalline regions separated by dislocated regions. Cellulose nanocrystals (CNCs) are ribbon-like nanoparticles that can be selectively isolated from the semicrystalline cellulose by hydrolyzing these dislocated regions using acids or enzymes. Depending on the source of the biomaterial and the hydrolysis conditions, CNCs will have well-defined sizes, aspect ratios, and crystal structure (*1*–*3*). Furthermore, these crystals display a high strength modulus (similar to steel), low abrasiveness, and low density at low cost.

A common acid used for the hydrolysis of CNCs is sulfuric acid. This reaction results in half-ester sulfate groups being grafted onto the surface of the crystals, producing a net negative charge in aqueous suspension. These surface charges allow the CNCs to form stable colloidal suspensions in water due to double layer electrostatic repulsion (*4*). One of the major breakthroughs came when Gray and coworkers discovered that, under the right conditions, aqueous CNC suspensions self-align to form left-handed helical arrangements (*5*). Above a critical concentration, the CNCs start to spontaneously align, resulting in phase separation into a lower chiral nematic phase and an upper isotropic phase. The lower chiral nematic phase fraction increases with increasing concentration, until it encompasses the whole volume. As such, CNCs belong to the lyotropic class of liquid crystals, by which chiral nematic phases form in response to changes in concentration (*4*).

The chiral nematic phase is characterized by the pitch (*p*) of the helical alignment, which corresponds to a full turn of successive planes of the rods. For a CNC suspension, the pitch can be tuned within the range of micrometers to tens of micrometers (*6*–*9*). Furthermore, the chiral nematic phase can be preserved upon drying, resulting in iridescent films (*10*, *11*). The dried material acquires a photonic bandgap and a consequent iridescent color. The attractive optical properties of this self-assembled bioderived photonic crystal have been used in applications such as optical encryption, chiral templates, and optical sensors (*12*, *13*). The successful application of CNCs in photonic applications crucially relies on their controlled deposition into large domains, showing long range periodicity and uniform orientation (*14*). While the pitch decreases during the drying process, the initial pitch in the aqueous suspension has a direct influence on the resulting film. For this reason, accurately characterizing the pitch in suspension holds considerable importance (*2*, *4*, *15*).

Scattering techniques offer an accurate means to characterize the pitch in suspension (*16*–*18*). However, these techniques provide only an average value for the pitch and do not allow observing spatial defects or tracking local variations of the pitch. Polarized optical microscopy (POM) is often preferred for imaging and characterization due to its ease of use and availability. Despite its common practice, an accurate determination of pitch is effective only when the director is perpendicular to the optical axis, and surface effects on CNC alignment might affect the determined pitch due to limited light penetration depth. For dried films, scanning electron microscopy (SEM) and transmission electron microscopy (TEM) are commonly used, but they only provide surface information and are not suitable for visualizing liquid suspensions (*19*–*21*). All these imaging methodologies lack the capacity for sectional imaging necessary to render three-dimensional (3D) representations of the CNC chiral nematic liquid state. Given the inherent 3D architecture of liquid crystalline CNC phases, true 3D imaging techniques are expected to yield a more accurate depiction of their structural properties and variations and defects within the complete volume (*22*, *23*).

In this respect, nonlinear optical imaging provides several benefits over other conventional methods (*10*). In second-harmonic generation (SHG) microscopy, the narrow two-photon interaction volume in the focus of the objective provides an inherent confocal effect, enabling precise slicing of structures. These 2D slices can then be combined to create a rendered 3D representation for visualization and analysis. In addition, because there is less scattering of near-infrared (IR) light in out-of-focus planes, specimens can be visualized much deeper and experience less photo-bleaching. Last, the control of incident and detected light polarization allows further analysis of chirality, local structure, and domain orientations (*24*–*31*). Nonlinear optical imaging also offers complementary capabilities by simultaneously capturing multiple higher-order responses through spectral filtering. For example, two-photon fluorescence can be recorded alongside SHG signals, providing information about local density or potential impurities (*32*).

Inspired by the extensive work that has been done on collagen using SHG imaging, we propose a similar approach for the study of CNCs (*33*–*37*). Collagen, due to its parallel fiber alignment, serves as an ideal candidate for high-contrast SHG imaging. Similarly, the helical structure of CNCs and their developing chiral nematic order are anticipated to produce a comparable effect. To our knowledge, SHG imaging of the chiral nematic alignment of CNC suspension has never been reported.

Here, we conducted SHG imaging of the chiral nematic phase of cotton-based CNCs. The SHG images show strong periodic contrast that can be related to the helical pitch of the 3D liquid crystal phase, regardless of orientation. The validity of this assignation was established through the characterization of the predominant second-order tensor components of the system using polarization analysis. This analysis additionally allowed relating the observed response to the local alignment of the CNCs inside the liquid crystalline structure. Subsequently, 3D models illustrating the suspension morphology were rendered. This allowed us to apply a 3D discrete Fourier transformation (DFT) technique to achieve an efficient evaluation of the pitch throughout the entire volume of the 3D model.

## RESULTS

### SHG microscopy

SHG is a nonlinear optical phenomenon whereby a material generates light at twice the frequency of the incident radiation. In order for SHG to occur, within the electric dipole approximation, the material must be non-centrosymmetric. The nonlinear second-order polarization, $P_{i}^{\left(2\right)}$ within the electric dipole approximation, can be expressed as follows

$$P_{i}^{\left(2\right)}\left(2\omega \right)=\chi_{\mathrm{ijk}}^{\left(2\right)}E_{j}\left(\omega \right)E_{k}\left(\omega \right)$$ (1)

where $\chi_{\mathrm{ijk}}^{\left(2\right)}$ is the second-order susceptibility tensor and *E_j_*(ω) and *E_k_*(ω) are the electric field components of the incoming light with frequency ω (*24*). The indices *ijk* refer to the Cartesian coordinates of the system frame. The measured SHG intensity, *I*_2ω_ can, therefore, be expressed as follows

$$I_{2\omega }∼{\left|\chi^{\left(2\right)}\right|}^{2}I_{\omega }^{2}$$ (2)

where *I*_ω_ is the intensity of the incoming light. It follows from Eq. 2 that, for a given crystal and for an excitation beam of fixed intensity, variations in intensity are directly related to the orientation of the crystals relative to the optical axis. The projection of the local components to the macroscopic framework can be described by three Euler angles: ϕ, θ, and Ψ. One could also consider contributions of second- and third-order nonlinear optical effects originating from water molecules and the static electric field of the CNC (*38*–*40*). This consideration would modify Eq. 2 by the addition of a third-order term [χ^(3)^*E_DC_*]^2^ and a mixing term 2χ^(2)^χ^(3)^*E_DC_* (*41*, *42*). However, because of the small Debye length and the resulting low interaction volume of water molecules, the third-order term can be neglected (*43*). Furthermore, the close proximity of the CNC, which is in the order of tens of nanometers (*18*), results in centrosymmetry, allowing the mixing term to be neglected as well from its second-order dependence. A detailed calculation of the Debye screening length and their effect on the second-harmonic intensity is detailed in text S16 and text S17, respectively (*42*, *44*–*50*).

### SHG polarimetry

SHG polarimetry is used to characterize the structural origin of the second-order response, determined by the relative contributions of the $\chi_{\mathrm{ijk}}^{\left(2\right)}$ tensor components. As laid out below, this analysis will allow us to relate the observed periodic variation in SHG intensity to the pitch, assign the structure of the chiral nematic phase to the *C*_2_ point group, and make an estimate of the natural deviations of the alignment of the CNCs.

**Fig. 1.**
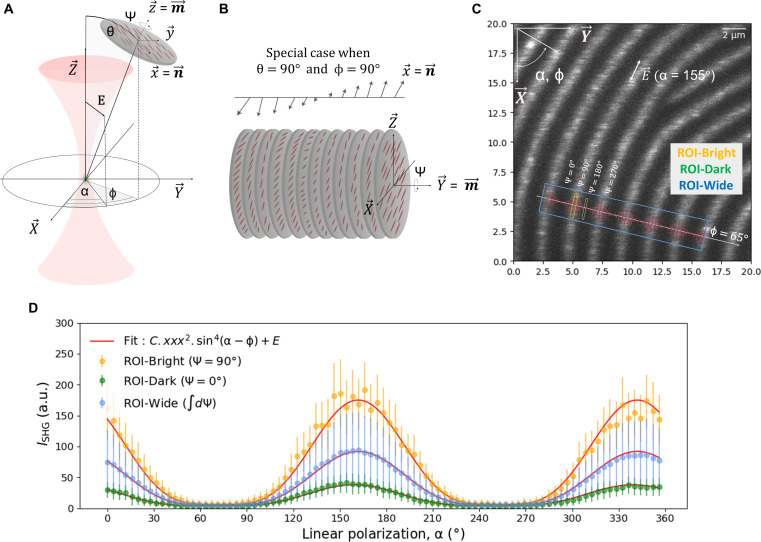
Polarization response in line with CNC orientation. (**A**) Mathematical framework used to describe the chiral nematic phase of CNCs using three azimuthal angles: Ψ, θ, and ϕ. The director, **n**, and the chiral axis, **m**, are in line with the *x* axis and the *z* axis of the molecular frame (*xyz*), respectively. The in-plane linear polarization angle is defined as α, rotating within the focal plane *XY* of the macroscopic frame (*XYZ*). (**B**) An illustration of the chiral nematic alignment for the special case where θ = 90° and ϕ = 90°. In this instance, the chiral axis, **m**, aligns with the *Y* axis of the focal plane *XY*. (**C**) An SHG image of 20 μm by 20 μm whereby the chiral axis lies within the focal plane, θ = 90°; the in-plane director is characterized by Ψ, which maps the pitch within the focal plane *XY*; and the orientation of the chiral axis **m** is characterized by ϕ. Polarization analysis is performed with incremental polarization steps of 4° with the current SHG image shown here corresponding to α = 155°. (**D**) Polarization patterns are observed in three specific regions of interest (ROIs), denoted as ROI-Bright, ROI-Dark, and ROI-Wide. The intensity profile correspond to the average intensity of the ROIs around Ψ = 90°, Ψ = 180°, and a broader viewing field that encompasses seven bright fringes, respectively. In these plots, the response is modeled on the basis of a purely polar *xxx* system, exhibiting a sin^4^ dependence with respect to linear polarization. The three responses show differences in intensity values with ROI-Bright displaying the highest intensity and ROI-Dark displaying the lowest intensity. Nevertheless, the profile of the responses remained consistent across all ROIs. The fitting is improved when probing a wider field of interest, as is the case in ROI-Wide, due to averaging of the signal combining both bright and dark regions. a.u., arbitrary units.

A model of the chiral nematic phase is shown in Fig. 1A. The structure is characterized by long range ordering of the CNCs whereby the director (**n**) rotates around the helical axis, **m** (*11*). The director, **n**, defines the average molecular orientation in each spiral staircase plane, and a full rotation of the director over a distance *p* corresponds to the pitch of the chiral nematic phase. **m** and **n** can be related to the macroscopic frame by Euler angles θ, Ψ, and ϕ, while the in-plane angle for the linear polarization of the laser light is described by α. Representing the chiral nematic phase, a Cartesian frame is defined, showing the helical axis (**m**) as well as the director (**n**) rotating around **m**. The helical axis **m** corresponds to the *z* axis in the molecular frame. The pitch angle, describing the orientation of the director around the helical axis, is related to Ψ, while the azimuthal angle of **n** corresponds to ϕ. The polar angle, θ, determines the out-of-plane orientation of the helical axis **m** relative to the *XY* focal plane. At θ = 90°, the helical axis **m** aligns with the focal *XY* plane as illustrated in Fig. 1B. In this case, a full rotation of pitch angle Ψ will map onto the *XY* focal plane as a periodic variation in intensity observed as fringes as shown in Fig. 1C.

**Fig. 2.**
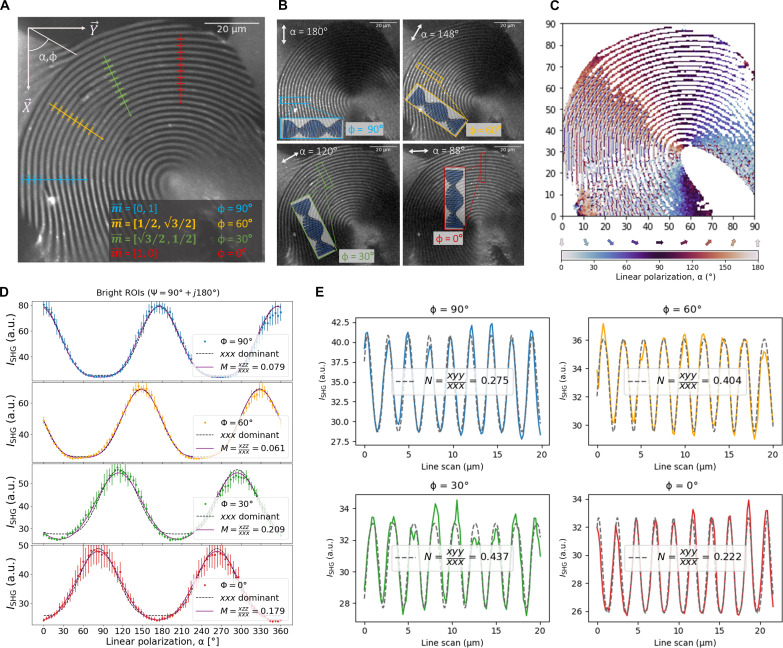
Phase response and fitting off-axis contributions. (**A**) SHG polarimetry was performed at 90 μm by 90 μm for a total of 91 images obtained through incremental rotation of 4° of the linear polarization. The recombined image from the intensity average of all of the 91 images. Four ROIs were defined at different chiral axis ϕ = 90°, 60°, 30°, and 0°. (**B**) Four images wherein each ROI was the brightest, the direction of linear polarization for each of these images is also included. A visual inspection of these results indicate that the polarization is perpendicular to the chiral axis (α = ϕ ± 90°) (**C**) Color map illustrating polarization degree α equivalent to the maximum *I*_SHG_ at each position. (**D**) Polarization patterns for four ROIs (each at ψ = 90°) with fits to *xxx* dominant response and the fit equation for *M* = *xzz*/*xxx*. The ROI responses are averaged from 10 narrow bright regions of along each chiral axis. (**E**) Line scan patterns of the four ROIs of the cumulative polarization signal and with fitting to determine *N* = *xyy*/*xxx*. Parameters $M=\frac{\mathrm{xzz}}{\mathrm{xxx}}$ and $N=\frac{\mathrm{xyy}}{\mathrm{xxx}}$ represent contributions of the additional off-axis components *xzz* = *zxz* and *xyy* = *yxy*.

A distinct region showing a radial cholesteric phase, shown in both Figs. 1 and 2, was selected for polarization analysis (*15*). This naturally occurring circular periodic morphology, resembling a fingerprint pattern, provides an ideal configuration for probing the polarization response for different orientations of the helical axis. It is noteworthy that, throughout the entire pattern, the distance between the observed fringes remains uniform, independent of orientation (see text S3). This implies that θ is near to 90° throughout the images, because any tilt in the plane containing the fingerprint pattern would result in local variations in the observed pitch due to differences in projection onto the *XY* plane. This assignment (θ = 90°) will simplify the tensor analysis presented below.

We found that the structure exhibits a response typically expected for polar structures. The SHG polarization patterns overall fit well to a response governed by a single dominant polar *xxx* component directed along **n**, following

$$I_{\text{SHG}}=\mathrm{Axx}x^{2}\text{si}n^{6}\left(\Psi \right)\text{si}n^{4}\left(\alpha -\phi \right)+E$$ (3)

Three regions of interest (ROIs), highlighted in Fig. 1C, were selected for fitting to Eq. 3. The respective polarization responses are plotted in Fig. 1D. The interpretation of the second-order response for this assignment is intuitive. For pitch angle Ψ = 90°, the director is in the *XY* plane. This results in a maximal response when aligned with the polarization angle, i.e., when the chiral axis is perpendicular to the polarization, α = ϕ ± 90°. Conversely, the response falls to zero when the director is perpendicular to the incident polarization. This occurs for Ψ = 0°, and, when the chiral axis is in line with the polarization angle, α = ϕ. The polarization response simulated for a single dominant polar response is provided in text S5. Using Eq. 3, an analysis of the local orientation of the chiral axis, corresponding to a phase shift ϕ, can be performed. Figure 2A shows an SHG image of a larger raster scanning area (90 μm by 90 μm). The in-plane polarization angles α and the in-plane orientation of the chiral axis ϕ are indicated in the *XY* reference frame. Four ROIs are selected for different values of ϕ, and inspection of the local intensity for different directions of the in-plane polarization α confirms that the maximum response occurs when the polarization is perpendicular to ϕ, as expected from Eq. 3. It follows from these polarization images that the dominant SHG response, corresponding to the polar *xxx* component, corresponds to the longitudinal axis of the CNCs, i.e., the director, **n**.

Upon closer inspection of the polarization patterns in Fig. 2D, small deviations from the expected sin^4^ dependence become apparent for some domains. This suggests that the SHG response contains additional contributions. This observation is in line with similar studies carried out by Schanne-Klein and coworkers, who investigated collagen cholesteric liquid crystals with SHG microscopy (*37*, *51*, *52*). They found similar periodic fringes in the focal plane. However, to adequately describe the nonlinear optical response, the authors had to introduce an additional off-axis component *zxx* alongside the dominant *xxx* component, in line with the *C*_∞*V*_ symmetry of the cholesteric phase. In the case of CNCs, the inherent *C*_2_ symmetry found in the individual CNC building blocks that make up the structure was chosen to analyze the SHG response. Under the assumption of Kleinman symmetry, which is valid because we measure far away from (one- and two-photon) absorption of the CNCs, two off-axis components *xzz* = *zxz* and *xyy* = *yxy* are introduced. The tensor describing the second-order susceptibility for this point group is known (*53*) and can be expressed as follows

$$\begin{array}{cc} \chi_{\mathrm{ijk}}^{\left(2\right)} & =\left(\begin{array}{cccccc} \mathrm{xxx} & \mathrm{xyy} & \mathrm{xzz} & 0 & 0 & 0\\ 0 & 0 & 0 & 0 & 0 & \mathrm{xyy}\\ 0 & 0 & 0 & 0 & \mathrm{xzz} & 0 \end{array}\right)\\ & =(\begin{array}{cccccc} \mathrm{xxx} & \text{Nxxx} & \text{Mxxx} & 0 & 0 & 0\\ 0 & 0 & 0 & 0 & 0 & \text{Nxxx}\\ 0 & 0 & 0 & 0 & \text{Mxxx} & 0 \end{array}) \end{array}$$ (4)

Parameters *M* = *xzz*/*xxx* and *N* = *xyy*/*xxx* represent contributions of two additional off-axis components *xzz* = *zxz* and *xyy* = *yxy*. Remark that chiral components can be neglected in this case, as laid out in the text S7. Solving Eq. 1 using $\chi_{\mathrm{ijk}}^{\left(2\right)}$ from Eq. 4 allows the derivation of general fit equations to determine *M* and *N*, which are provided in the text S8 (eqs. S13 and S14). We find that *M* and *N* can be singled out conveniently when the helical axis **m** lies in the imaging plane, as is the case in the fingerprint pattern. In this case, a value for *N* can be determined from a fit to the line profile, while a value from *M* follows from the polarization pattern. Fits for the different ROIs are shown in Fig. 2 (D and E), while the derived values for *M* and *N* are given in Table 1. We observe that *M* and *N* exhibit considerable variability across various domains. These variations can be attributed to differences in the local structure of the chiral nematic phase, due to changes in the alignment of the CNCs. Stated otherwise, the variation in the macroscopic off-axis components *M* and *N* contains information about the local distribution of the CNCs.

**Table 1.** Summary of the fitted *M* and *N* and their respective aperture angles. The four ROIs correspond to the ones shown in Fig. 2. The error propagation for the angles originates from the error of the nonlinear fitting and is expanded in detail in text S10.

Chiral axis: ϕ	$M=\frac{\text{xzz}}{\text{xxx}}$	$N=\frac{\text{xyy}}{\text{xxx}}$	$\langle \frac{\sigma_{1,0}}{2}\rangle$	$\langle \frac{\sigma_{2,0}}{2}\rangle$
90°	0.079 ± 0.018	0.275 ± 0.018	30.8° ± 6.1	28.2° ± 2.8
60°	0.061 ± 0.015	0.404 ± 0.013	34.3° ± 5.9	21.2° ± 2.4
30°	0.209 ± 0.015	0.437 ± 0.022	38.8° ± 2.7	34.7° ± 1.2
0°	0.179 ± 0.012	0.222 ± 0.024	32.3° ± 3.8	41.9° ± 1.8

**Fig. 3.**
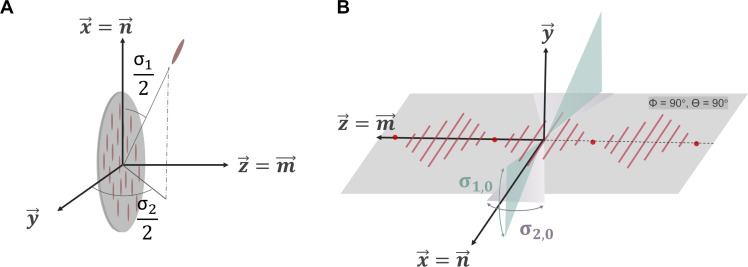
Aperture angles represent deviations from perfect CNC. (**A**) Each stack in the CNC alignment can be interpretated as an ensemble of one-dimensional SHG active individual molecules with an angular distribution defined by aperture angles $\frac{\sigma_{1}}{2}$ and $\frac{\sigma_{2}}{2}$. (**B**) The aperture angles are the result from the integration of the angular distributions and are denoted $\frac{\sigma_{1,0}}{2}$ and $\frac{\sigma_{2,0}}{2}$. The aperture angle σ_1,0_ is in the *xy* plane along the helical axis, while σ_2,0_ represents the aperture angle along the director.

To relate the supramolecular response measured by *M* and *N* to the orientational distribution of the CNCs, the second-harmonic response of individual CNCs must be known. This response is described by the various elements β*_ijk_* of the CNC hyperpolarizability tensor. As a first approximation, a single dominant β*_xxx_* component can be assumed. This assumption is strengthened by noting that macroscopic off-axis components are minimal in some domains, contributing less than 5% in certain cases, thus emphasizing the dominance of this component. Analogous to other fibrillar structures such as collagen, an angle representing the width of the distribution of the CNCs with respect to the supramolecular framework can then be derived by performing an appropriate coordinate transformation, as represented in Fig. 3A (see text S9) (*27*, *54*). For CNCs, their local alignment can be conceptualized as a distribution along helical axis **m** in the *xz* plane, related to *M*, and along director **n** in the *yz* plane, related to *N*. Two aperture angles representing the width of the distribution in both directions are derived as follows

$$M=\frac{\mathrm{xzz}}{\mathrm{xxx}}={\text{tan}}^{2}\left(\frac{\sigma_{1,0}}{2}\right).{\text{sin}}^{2}\left(\frac{\sigma_{2,0}}{2}\right)$$ (5)

$$N=\frac{\mathrm{xyy}}{\mathrm{xxx}}={\text{tan}}^{2}\left(\frac{\sigma_{1,0}}{2}\right).{\text{cos}}^{2}\left(\frac{\sigma_{2,0}}{2}\right)$$ (6)

$$\frac{M}{N}=\frac{\mathrm{xzz}}{\mathrm{xyy}}={\text{tan}}^{2}\left(\frac{\sigma_{2,0}}{2}\right)$$ (7)

The aperture angles related to the distribution of the CNCs are represented graphically in Fig. 3B. Table 1 summarizes the fitted values for *M* and *N* and the derived aperture angles across different domains. While the aperture angles should be regarded as an estimate, it is apparent that the distribution of the CNCs is anisotropic. The aperture angle $\frac{\sigma_{1,0}}{2}$ in the *xy* plane along the director **n** of the chiral nematic phase is estimated to vary between 31° and 39°. In contrast, in the *xz* plane, a larger variation in angle $\frac{\sigma_{2,0}}{2}$ along the helical axis **m** is apparent depending on the specific ROIs, ranging between 21° and 42°. We suspect that this variation arises from the circular twisting of the helical axis in the fingerprint pattern, potentially causing additional localized strain on the CNCs.

### 3D imaging and DFT characterization of the helical pitch

In the next section, we will detail how SHG imaging can be used to obtain insights into the 3D helical structure of the chiral nematic CNC phase. To proceed, we first need to establish an unambiguous relation between the observed periodic variations in SHG intensity and the helical pitch. Starting from the tensor components of the *C*_2_-symmetric chiral nematic CNC phase from Eq. 4, we performed a theoretical analysis (text S6), confirming that the pitch corresponds to the spacing between the points of maximum intensity in the SHG image, as long as the relative contribution of the off-axis components remains below 50%. The values that we determined fall well within this range (Table 1). For more substantial off-axis contributions (>50%), additional fringes appear when using linearly polarized light, disrupting the direct link between intensity and pitch. We found that such artifacts can be avoided by using circular incident polarization (text S12). The samples measured by 3D *z*-stacking were, therefore, imaged using circular polarization illumination, which has the additional benefit that the SHG intensity no longer depends on the orientation of the chiral axis in the focal plane (i.e., ϕ).

**Fig. 4.**
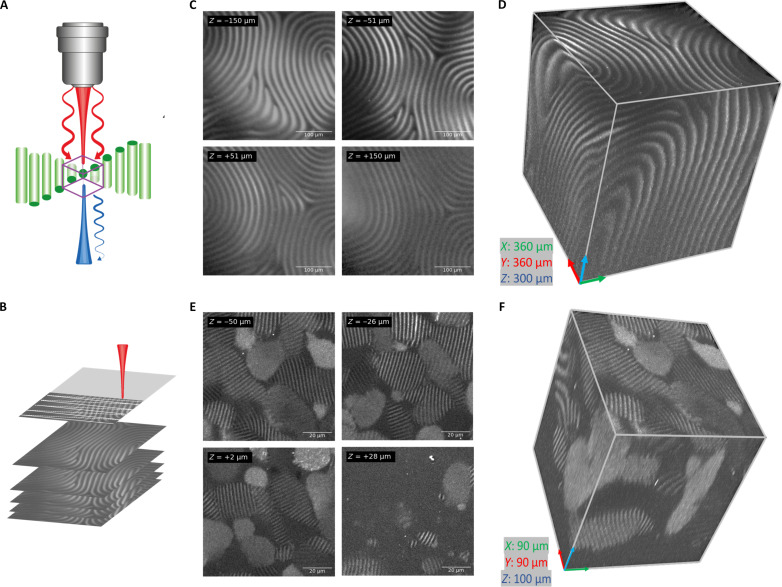
Focus stacking 3D structures. (**A**) Illustration of maximum SHG response whereby the director is perpendicular to the optical axis and how raster scanning within the structures renders differences in contrast. (**B**) Z-stacking working principle illustrating the raster scanning of the image and the stacking of each individual slice. (**C**) Four slices of a total of a 101 acquired slices for the *z*-scanning of chiral nematic CNC at 8 wt % in a 2-mm quartz cuvette. The step size in the *Z* direction is 3 μm. The *XY* dimensions of each 2D slice are 360 μm by 360 μm. (**D**) 3D representation has dimensions (*XYZ*) of 360 μm by 360 μm by 300 μm. (**E**) Four slices of a total of a 51 acquired slices for the *z*-scanning of chiral nematic CNC at 2 wt % in a thin 716-μm cuvette. The step size in the *Z* direction is 2 μm. The *XY* dimensions of each 2D slice are 90 μm by 90 μm. (**F**) 3D representation has dimensions (*XYZ*) of 90 μm by 90 μm by 100 μm.

The strong SHG contrast resulting from the helical twisting of the director, combined with the inherently confocal effect of two-photon generation, allows for highly resolved 3D imaging of the micrometric periodicity of the chiral nematic CNC phase. Figure 4 (A and B) illustrates the focus stacking technique that involves taking multiple images with incremental focal plane steps and recombining them into a 3D model of the structure. The *z*-resolution is ~2.5 μm (*34*). During the *z*-scanning, the step sizes are closely matched to this resolution, whereby, in Fig. 4 (C and E), 3 μm per slice and 2 μm per slice were chosen, respectively.

As can be seen in Fig. 4, 3D imaging offers a powerful visual representation of the chiral nematic phase. The technique allows tracking the pitch in 3D space and provides insight into the local domain orientations within the suspension. In this manner, defects and domains are readily revealed. In Fig. 4C, a continuous defect is observed along the entire acquired volume, which can be identified as a twist grain boundary (*55*). In Fig. 4D, several smaller domains are observed that have coalesced together to form a fused network of smaller-ranged alignments. In the uppermost slices of Fig. 4D, we enter the isotropic phase and therefore periodicity is no longer observed. An SHG signal resulting from the CNCs remains detectable. It is also worth pointing out that, even within the chiral nematic phase, some regions show no observable pitch. A possible explanation for this could be that these domains have their helical axis (**m**) parallel to the optical axis of the objective. In this orientation, the twisting of the director would no longer result in variation of the SHG intensity for circular polarization, and the periodicity would, therefore, no longer be observed. Moreover, depending on the pitch length, the more limited *z*-resolution may not be able to resolve the pitch in this configuration, even for linearly polarized light. That being said, domains that are almost parallel to the optical axis are still observed in Fig. 4D. Therefore, the aforementioned limitation would only occur in very limiting cases. Another possible explanation for the absence of measurable periodicity could be high hydrodynamic shearing forces within the tight confinement geometry that would disrupt the CNC alignment (*22*). High shearing forces that untwist the helical structures into parallel ones have already been observed in the literature (*16*, *56*). This opens perspectives to study this phenomenon in situ with SHG microscopy.

Last, the high contrast of the SHG images is ideally suited for performing DFT. DFT is a mathematical technique that can be used to analyze the frequency components of a signal and is commonly used for characterizing periodicity in images. Because of the clear distinction between the bright and dark regions in the SHG images, DFT provides clear peaks in the spectral domain that can be identified accurately. *^p^*/_2_ is then obtained by taking the inverse of the nonzero maximum wave vector of the radial profile. If the spectral domain has been calibrated to the physical dimensions of the image, then the pitch can be obtained from

$$\frac{p}{2}=\frac{1}{k_{\text{max}}}$$ (8)

Using DFT to measure pitch offers several advantages over manual profile plots, as is common in literature. It allows deriving an average pitch that takes into account and the entire volume and all directions simultaneously. Moreover, scripting can be applied to data processing, ensuring more reproducible and efficient calculations, particularly when dealing with a large number of samples.

**Fig. 5.**
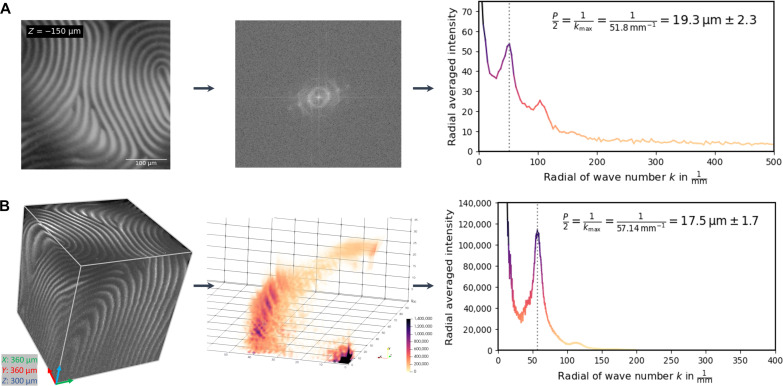
2D-DFT and 3D-DFT for pitch measurement. (**A**) The uppermost 2D SHG slice of the *z*-stacking with dimensions of 360 μm by 360 μm (*XY*) with the respective 2D-DFT *k*-space and radial profile. The results for the 2D-DFT of the pitch value is 19.3 μm. (**B**) The reconstructed 3D image with dimensions of 360 μm by 300 μm by 300 μm (*XYZ*) with the respective 3D-DFT and radial profile. The results for the 3D-DFT of the pitch value is 17.5 μm. The calculation for the SDs of the pitch is taken from the full width at half maximum (FWHM) of the peak profiles.

DFT is applicable in both 2D and 3D imaging (2D-DFT and 3D-DFT). In Fig. 5A, the 2D-DFT of the SHG image is provided, with the center of the image representing the DC field and all *k*-vectors extending outward. If the chiral axis (**m**) only has a single direction, then the dominant *k*-vector would be brightest close to a point in line with the orientation of **m**. However, if the chiral axis has multiple orientations, as is the case here, then the 2D-DFT would manifest as concentric rings. The first ring corresponds to the *k*-vector of interest as the outer rings are harmonics of the first one. The average pitch, considering all directions, is obtained as the maximum of the radial profile of the 2D-DFT. Analogously to the 2D-DFT, the 3D-DFT corresponds to a sphere, with the center representing the DC field and all *k*-vectors extending outward relative to the chiral axis orientations. The 3D-DFT is illustrated in Fig. 5B and depicted as one-eighth of a sphere. By finding the maxima of the radial profiles in both the 2D and 3D, we can determine the pitch for both images. We observed a difference between the measured pitches of 19.3 μm in 2D against 17.5 μm in 3D. The value for the 3D measurement accounts for a greater amount of variation of pitch within the volume, but, crucially, it remains the same irrespective of the tilting of the domains. When the domains become misaligned with respect to the optical axis, the pitch measured in 2D will be larger than its true value. For these reasons, the 3D-DFT analysis offers a more accurate estimate of the pitch compared to conventional 2D imaging techniques. Furthermore, we cross validated the 3D-DFT by performing a line scan in tilted 2D projections from tomographic software and the results were consistent (text S13).

### Pitch measurements with varying CNC concentration

**Fig. 6.**
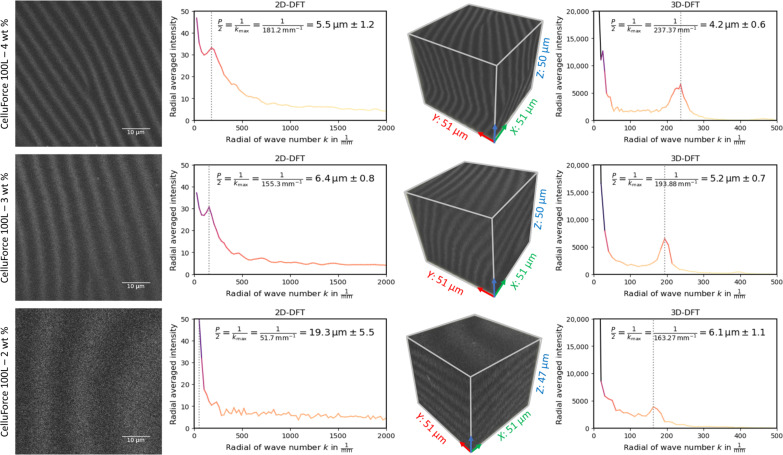
Pitch evolution with decreasing CNC concentration. *Z*-stacking of three samples of CelluForce 100L at 4, 3, and 2 wt % shown, respectively, in the top, middle, and bottom rows. The scanning area corresponded to 51 μm by 51 μm with a total of 51 slices. The raster scanning plane corresponds to the *XY* plane and is represented by the uppermost plane of the cube. The steps for the *z*-scan were fixed at 1 μm per slice, resulting in a total *z*-distance of 50 μm. In the left columns, a single 2D slice from the *z*-scanning is shown with the corresponding 2D-DFT. In the right columns, the reconstructed 3D model is shown with the corresponding 3D-DFT. The peaks of the DFT correspond to values for *^p^*/_2_ of 5.5, 6.4, and 19.3 μm in 2D, compared to 4.2, 5.2, and 6.1 in 3D, with decreasing concentration. The differences originate from the tilting of the chiral nematic domains, with the 3D value providing more accurate physical quantification. The calculation for the SDs of the pitch is taken from the FWHM of the peak profiles.

To validate the aforementioned technique, a widely recognized standard of sulfated CNC suspension, CelluRods 100L (CelluForce) was used. The material demonstrated a high degree of alignment without any observable isotropic phase. Suspensions of 2, 3, and 4 wt % were prepared, and their pitch was characterized using the *z*-scanning method. All three diluted suspensions showed the presence of a biphasic regime. The results of the scans, covering a volume of 51 μm by 51 μm by 50 μm (*xyz*), of the CelluForce samples are provided in Fig. 6. 3D-DFT was performed on the three volumes of interest, and the maxima of the radial profile of their respective *k*-space were then used to derive the pitch dimensions.

As anticipated based on existing literature, a reduction in pitch was observed with increasing concentration. For the CelluForce at 4 wt %, a pitch of 4.2 μm was measured, compared to 5.2 μm for the CelluForce at 3 wt % and 6.1 μm for the CelluForce at 2 wt %. It is important to highlight that these pitch measurements derived from the 3D stacks yield smaller values compared to those obtained from the 2D stacks. 2D projections of tilted domains result in apparent pitch dimensions that are greater than their actual physical dimensions. This phenomenon, while true for all samples, is most pronounced in the 2 wt % sample for which the chiral nematic exhibits a pronounced tilted orientation with respect to the optical axis. For this reason the *XY* slicing of the structure reveals a substantially longer pitch than what is measured in 3D (19.3 μm versus 6.1 μm). With POM highly tilted domains could not be observed. Last, polarimetry analysis to assess natural variations was conducted in CelluForce at 3 and 4 wt %. The calculated aperture angles are detailed in text S11 and are comparable to the values obtained for the 2 wt % cotton-based sample (see Table 1).

## DISCUSSION

Our study details groundbreaking work on the label-free visualization and characterization of the chiral nematic phase of CNC suspensions using SHG microscopy. The supramolecular organization of chiral CNC nanoribbons produces strongly contrasted SHG images, offering unprecedented insights into the micro-periodic structure of the liquid crystalline phase. Through an in-depth analysis of the second-order nonlinear optical response, we were able to relate the SHG response to the orientational distribution of the CNC nanoribbons. This revealed local differences in alignment of CNCs between different directions of the liquid crystalline lattice. Next, we demonstrated a direct relation between the observed variations In SHG intensity and the helical pitch. This allowed us to capture the first successful 3D images of the helical structure of chiral nematic CNCs through focus *z*-stacking, revealing intricate details about their helical pitch, polycrystallinity, and the occurrence of defects. The high-resolution images that we have acquired provide a level of detail that has not been seen before. Furthermore, the high contrast of the SHG images facilitated a 3D Fourier analysis, which allowed a more accurate determination of the pitch in these systems. When applied to a concentration series, the 3D-FFT method proved to be more accurate than the traditional 2D determination of pitch, particularly for tilted samples where the difference became more pronounced.

Building on this work, our research not only advances the understanding of CNC chiral nematic systems but also sets the stage for broader application. Much like the capability to image the structure of collagen through SHG microscopy sparked a very active field of research, we believe that our work has the potential to catalyze a similar surge of interest and exploration within the cellulose community. We are convinced that the techniques and insights that we have introduced can be applied to a wide range of cellulose systems, from liquid crystalline phases to dried films, and composite materials. Future work is directed at using our methodology to validate strategies aimed at achieving controlled deposition of photonic CNC structures in aqueous and dried conditions. We also plan to further explore the molecular second-order nonlinear optical responses of individual CNC rods via theoretical modeling to evaluate their macroscopic alignment through SHG imaging with increased precision.

## MATERIALS

### Products

Cotton wool was purchased from Hartmann AG (Germany). Sulfuric acid (95%), Dowex Marathon Na-form, and Dowex Marathon H-form ion exchange resins were obtained from Fisher Chemicals. Spectra/Por type 3 [molecular weight cutoff (MWCO) of 3 to 5 kDa] and type 4 (MWCO of 16 to 18 kDa) dialysis tubing membranes were obtained from SpectrumLabs. Standard solutions for conductometric titration (0.01 M sodium hydroxide and conductivity standard of 1413 μS/cm) were obtained from ChemLab NV. All products were used as received. CelluForce 100L was ordered from CelluForce and used to prepare a concentration series (4, 3, and 2 wt %). The suspension was dialyzed against deionized water of effluent water for a period of 24 hours and then used as is.

### Preparation of CNC suspension

The CNCs were prepared according to a standard procedure for the hydrolysis of raw cellulose using 64 wt % sulfuric acid (*1*). Cotton wool was hydrolyzed at elevated temperature (45°C, 45 min, cotton:acid ratio = 1:9), centrifuged at 8000 rpm for 20 min at 4°C until the pH of the supernatant reached 1, and dialyzed against deionized water until neutrality of the effluent water. The suspension of the sulfated cotton CNCs was sonicated on a Bandelin high-performance sonifier, filtered through a glass pore filter (pore size of 2), and treated with Dowex Marathon resin in an ion exchange column. An H-form resin was used or preparation of an acidic suspension. The suspension was then concentrated with osmotic compression in a 20% PEG-2000 solution.

### Sample preparation for SHG microscopy

Two different sample preparations were used for SHG microspcopy, which differ by their containers. The confinement containers used were (i) 2-mm quartz cuvettes and (ii) resin encapsulated glass slides of 720 μm thickness. For the resin encapsulated glass slide, a Scotch tape of 720 μm thickness was used to encapsulate a cavity with a width of 3 mm and a length of 10 mm. A microslide was cut to fit and added on top of the Scotch tape. The required amount of ultraviolet (UV) resin was added to the contour of the Scotch tape to encompass the glass slide. The deposited resin was cured with a UV lamp for 5 min to hermetically seal the CNC suspension without coming in direct contact with the latter. The aim of the UV preparation was to retain the water content in the suspension while reducing the optical path and the total volume of the container. The total volume of this confinement geometry was 22 μl in comparison to the 700 μl of the quartz cuvette. The samples used for each analysis are summarized in Table 2.

**Table 2.** Summary of sample preparation.

Source	Concentration	Container	Volume	Optical path	Polarimetry performed?	3D stacking performed?
	(wt %)		(μl)	(μm)		
Lab preparation	8.0	Quartz cuvette	700 μl	2000 μm	No	Yes
Lab preparation	2.0	Thin slide	22 μl	720 μm	No	Yes
Lab preparation	8.0	Thin slide	22 μl	720 μm	Yes	No
CelluForce 100L	4.0	Thin slide	22 μl	720 μm	Yes	Yes
CelluForce 100L	3.0	Thin slide	22 μl	720 μm	Yes	Yes
CelluForce 100L	2.0	Thin slide	22 μl	720 μm	No	Yes

An observation that should be mentioned is the notable differences in the measured pitch between the polarimetry and the *z*-scanning. The values for *^p^*/_2_ in the *z*-stacking were approximately one order of magnitude greater than the ones observed for the polarimetry (17.5 μm against 2.5 μm). While these substantial differences were unexpected, they can be attributed to the sample preparation process. The polarimetry analysis used a thin-slide preparation, subjecting the suspension to higher shearing forces. These forces originate from the action of pressing the cover slip onto the suspension and sealing the volume at all edges, thus impeding any movement (*57*). For this reason, the small confined volume within the thin slide experienced much stronger geometrical constraints compared to the free resting suspension of the *z*-scanning, found in 2-mm quartz cuvette.

### Laser and optical layout

The laser used was a mode-locked pulsed laser (Insight DS+ Spectra-Physics) producing a horizontally polarized beam of 80-MHz repetition rate and a 120-fs pulse duration. SHG is a nonresonant process so the choice of the wavelength is not critical in terms of signal intensity as the ß decreases slowly with increasing wavelength. However, the excitation of 900 nm was chosen to allow sufficiently large penetration depths while keeping the output power high and the pulse duration short. The total output power of the laser was 1.2 W at 900 nm with a beam diameter of 1.2 mm. The setup was mounted on an anti-vibration table. The setup was the same as described in the work by de Coene *et al.* (*42*).

### Power and polarization

The laser power was modulated by a combination of a half-wave plate and a Glann-Taylor polarizer. The linear horizontally polarized light is rotated by the achromatic half-wave plate and split into its vertical and horizontal components by the Glann-Taylor polarizer. Only the subsequent vertical polarization was retained in the beam path with the horizontally polarized component being beam dumped. The rotation, through motorized motion control, of the half-wave plate allowed superior and faster control of the excitation power than the laser source because the latter only offers limited dynamic range. Furthermore, the polarization method for power control induces no alignment issues because the beam path remains undeflected by the optical components. The illumination power can be expressed from the following quadratic cosine equation

$$P=P_{\text{max}}{\left[\text{cos}\left(2\theta \right)\right]}^{2}$$ (9)

The power used for the imaging and the polarization was the same with the angle of the first λ/2 set to θ = 59° that translated to a theoretical value for power of 265 mW.

The polarization state of the beam before the scanning system was circular for the 3D imaging and linear for the polarization analysis. In the case of 3D imaging, a quarter-wave plate is positioned before the scanning system at 45° to induce circular polarization. The quarter-wave plate was controlled through motion control and rotated by an offset from 45° to compensate for the elliptical scrambling of the scanning system. This correction was carried out from the live images of the sample to tweak the circular polarization at focus. In the case of the polarization analysis, a half-wave plate was positioned before the scanning to induce rotation of the linear polarization across successive image acquisitions.

### SHG polarimatery setup

To describe the electric field components from the laboratory frame (*E_X_* and *E_Y_*) to the system frame (*E_x_*, *E_y_*, and *E_z_*), we need to perform a transformation using the rotational transformation matrix, **a**

$$\left[\begin{array}{c} E_{x}\\ E_{y}\\ E_{z} \end{array}\right]=a\left[\begin{array}{c} E_{X}\\ E_{Y} \end{array}\right]$$ (10)

The transformation matrix is a 3 × 2 matrix because we assume that there is no electric field in the optical axis (i.e., *E_Z_* = 0). This assumption is valid for objectives with a numerical aperture (NA) of up to 0.8 (*58*). The transformation matrix contains all the Euler angles of the transformation. Applying the above relation to Eq. 1 provides the nonlinear polarization components $P_{x}^{\left(2\right)}$, $P_{y}^{\left(2\right)}$, and $P_{z}^{\left(2\right)}$ in the system frame. We can then apply the reverse transformation of $P_{x}^{\left(2\right)}$, $P_{y}^{\left(2\right)}$, and $P_{z}^{\left(2\right)}$ back to the laboratory frame that delivers the polarization components $P_{X}^{\left(2\right)}$ and $P_{Y}^{\left(2\right)}$. These can then be directly related to the measured quantities *I_X_* and *I_Y_*

$$I_{X}∼{\left|P_{X}^{\left\{(2)\right\}}\right|}^{2}$$ (11)

$$I_{Y}∼{\left|P_{Y}^{\left\{(2)\right\}}\right|}^{2}$$ (12)

**Fig. 7.**
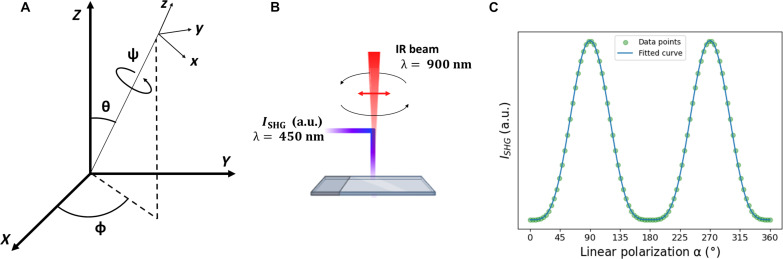
SHG polarimetry scheme. (**A**) The relative orientation of the system frame (*xyz*) with regard to the laboratory frame (*XYZ*) is expressed by means of the Euler angles Φ, θ, and Ψ. (**B**) The excitation beam operates at a wavelength of 900 nm, and the resulting SHG signal is detected at 450 nm in all orientations. During the polarization analysis, the linear polarization is varied incrementally between successive images. The overall SHG signal generated is measured in reflection mode. (**C**) An example of a polarization analysis response involving the acquisition of 91 images. In this example, the linear polarization, α, is incremented by 4° in between each successive image. The graph also shows a mathematical fit applied to these specific data points.

The quantities *I_X_* and *I_Y_* can be measured directly by adding an analyzer in the detector path. In the absence of an analyzer, the total intensity is found as the sum of these quantities. A more detailed description of the transformation matrices can be found in the Supplementary Materials (text S1). On the basis of this formalism, information about the contributing $\chi_{\mathrm{ijk}}^{\left(2\right)}$ tensor components can be retrieved. Because symmetry elements in the structure of the sample have the effect of reducing the number of nonzero independent components $\chi_{\mathrm{ijk}}^{\left(2\right)}$, information about the (point group) symmetry of the sample can be retrieved by measuring the second-order nonlinear optical susceptibility tensor (*59*). Polarized tests are commonly used to probe the different elements of $\chi_{\mathrm{ijk}}^{\left(2\right)}$ (*4*). In this work, we conducted a polarization series by incrementally changing the incoming linear polarization angle, α, and imaging the chiral nematic phase by SHG microscopy. This is further detailed in Fig. 7.

### Microscope and objectives

High-NA water immersion objectives were used for both imaging and polarization tests. For the imaging analysis and point group polarization analysis a 40× objective was used (NA, 0.70; working distance, 3.5 mm, Nikon). This objective offered optimized transmission in near IR with good spatial resolution. The long working distance of the objective was also favorable for avoiding contact when observing structures located deep within a bulk volume, for instance, the suspension in the quartz cuvette. Lateral resolution can be approximated by the diffraction limit which is λ/2 NA = 642 nm. However, because of the quadratic dependence of multiphoton, this should be considered as an upper limit and an improved resolution is expected. In a different paper, Campagnola and coworkers have demonstrated that, in a similar setup, using subresolution fluorescent beads excited at 800 nm with a 0.8-NA objective and optical resolutions of about ~700 nm laterally and ~2.5 μm axially could be obtained (*34*).

### Acquisition: Polarimetry

A half-wave plate was mounted on a motorized holder to control the angle of linear polarization. In all polarimetry experiments, a kinetic series of 90 images was run with no time offset between successive scans. The half-wave plate was rotated successively in between each image acquisition. The rotation of the λ/2 was carried out by increments of 2°, resulting in a change in linear polarization of 4°. This results in a total rotation of 360° of the linear polarization with image 1 corresponding to α = 0° and image 91 corresponding to α = 360°. In the polarimetry experiments, the SHG signal was collected in reflection mode.

Polarimetry was performed twice in the 8 wt % lab preparation suspension in the thin-slide preparation. The difference between both experiments lies in the use of different raster scanning zooms. The first experiment was performed at 18.5× raster zoom that corresponded to a field of view (FOV) of 20.02 μm by 20.02 μm, while the second experiment was performed at 4× raster zoom that corresponded to a FOV of 90.12 μm by 90.12 μm. The image size was 512 × 512 pixels with a sampling speed of 20 μs/pixel and “Line Kalman” averaging of 4. The total acquisition time for this polarimetry experiment was ~36 min.

Polarimetry was performed on 3 and 4 wt % CelluForce 100L. The differences between both these experiments also lie in the use of different raster scanning zooms. Because the pitch of the 4 wt % is smaller than the one for the 3 wt %, a higher raster scanning zoom was performed on the 4 wt %. Specifically, the zoom for 4 wt % was 7× that corresponded to a FOV of 51.5 μm by 51.5 μm, while the zoom for the 3 wt % was 5× that corresponded to a FOV of 72.1 μm by 72.1 μm. The images size was 512 × 512 with a sampling speed of 40 μs/pixel and Line Kalman averaging of 3. The total acquisition time for these polarimetry experiments was ~50 min.

### Acquistion: 3D imaging

A quarter-wave plate to generate circularly polarized light was added. SHG 3D imaging was performed on the 8 wt % lab preparation suspension in the 2-mm quartz cuvette. The acquisition was acquired at a 1× raster scanning zoom that corresponded to a FOV of 360.5 μm by 360.5 μm. The *z*-scan was performed with step size of 3 μm per slice with 101 slices for a total *z*-distance of 300 μm. The image size was 1024 × 1024 pixels with a sampling speed of 20 μs/pixel and Line Kalman averaging of 3. The total acquisition time for this z-scan experiment was ~1 hour and 46 min. The SHG signal was collected in reflection and transmission mode, and their grayscale values were averaged to reduce noise.

SHG 3D imaging was performed on the 2 wt % lab preparation suspension in the thin-slide preparation. The acquisition was acquired at a 4× raster scanning zoom that corresponded to a FOV of 90 μm by 90 μm. The *z*-scan was performed with step size of 2 μm per slice with 51 slices for a total *z*-distance of 100 μm. The image size was 1024 × 1024 pixels with a sampling speed of 20 μs/pixel and Line Kalman averaging of 3. The total acquisition time for this *z*-scan experiment was ~54 min. The SHG signal was collected in reflection and transmission mode, and their grayscale values were averaged to reduce noise.

SHG 3D imaging was performed on the CelluForce 100L at 4, 3, and 2 wt %. The acquisition settings were the same for all three samples. The raster scanning zoom was set to 7× that corresponded to a FOV of 51.5 μm by 51 μm. The *z*-scan was performed with a step size of 1 μm per slice with 51 slices for a total *z-*distance of 50 μm. The image size was 512 × 512 pixels with a sampling speed of 40 μs/pixel and Line Kalman averaging of 4. The total acquisition time for this *z*-scan experiment was ~1 hour. The SHG signal was collected in reflection and transmission mode, and their grayscale values were averaged to reduce noise.

### 3D visualization software

The 3D image rendering was performed with Fiji (ImageJ) and Zeiss Blue V2.2 (Lite). In ImageJ, the images are imported as an image sequence, and the brightness/contrast is adapted to normalize intensity between slices. *XY* dimensions are set by setting scale of 512 pixels to the FOV of the scans, and the *Z* dimension is set in properties with depth of the voxel set to the step of the *z*-scan. 3D visualization is performed with 3DViewer plugin of ImageJ with a scaling factor 1:1. The modified images have also been imported to ZenBlue Lite software (free version) for orthogonal display cuts.

The use of Fiji rendered convincing results for the 3D modeling; however, more advanced 3D software could also be used for optimizing the representations of these image stacks (*60*). For instance, sliced projections can be obtained in any arbitrary direction through the use of visualizing software. Orthogonal slices were generated by using Zeiss Blue software and provided in the Supplementary Materials as demonstration (text S13).

### 2D-DFT processing

Processing was carried out in Fiji (ImageJ) by performing an DFT of the image. A radial profile was performed on the azimuthal averaging of the DFT transform. The radial profile of the 2D DFT presented a peak average value for the pitch by combining all the directions in 2D space. The corresponding pitch is calculated from the following equation

$$\frac{p}{2}=\frac{\text{FOV}\;\text{of original image}}{\text{Radius}\;\text{at}\;\text{maximum intensity of}\;\text{DFT}}$$ (13)

### 3D-DFT processing

The 3D imaging data in Fourier space were analyzed using the NumPy and SciPy Python modules (*13*, *61*). The image data with brightness and contrast adapted to normalize intensities were read from a collection of 101 png files, one for each *z*-slice, at a resolution of 1024 pixels × 1024 pixels. The grid resolution ∆*x* = *Lx*/*Nx* = ∆*y* = *Ly*/*Ny* = 352.05 nm and ∆*z* = *Lz*/(*Nz *− 1) = 3 μm.

3D discrete cosine transform (DCT) of type II to the data as implemented in scipy.FFT.dctn was applied (*61*). The main advantage of using the DCT over the DFT is that it does not assume periodicity of the data across the frame boundary, i.e., the left and right sides are not the same side, and neither is the top/bottom and front/back side. The DCT is typically implemented using the DFT and, therefore, also takes advantage of its efficiency. As coordinates for the Fourier space, we use wave vectors **k** = λ^−1^, the inverse of the wavelength λ. The data returned by the DCT are sampled in bins of specific wave numbers. These bins have a size of Δ*k_x_* = Δ*k_y_* = 1.387 mm^−1^ and Δ*k_z_* = 1.650 mm^−1^ corresponding to the aforementioned resolutions in real space.
