# Supplementary material for: Imaging with a twist: Three-dimensional insights of the chiral nematic phase of cellulose nanocrystals via SHG microscopy
Source: Sci Adv. 2024 Oct 30;10(44):eadp2384. doi: 10.1126/sciadv.adp2384 (PMC11524189; doi:10.1126/sciadv.adp2384)
Supplement: Supplementary file 1 — Text S1 to S17 Figs. S1 to S16 Table S1 [file sciadv.adp2384_sm.pdf]

Science Advances
AAAS

Supplementary Materials for

Imaging with a twist: Three-dimensional insights of the chiral nematic phase of cellulose nanocrystals via SHG microscopy

Thibaut Legat *et al.*

Corresponding author: Stijn Van Cleuvenbergen, stijn.vancleuvenbergen@kuleuven.be;
Wim Thielemans, wim.thielemans@kuleuven.be

Sci. Adv. **10**, eadp2384 (2024)
DOI: 10.1126/sciadv.adp2384

This PDF file includes:

Text S1 to S17
Figs. S1 to S16
Table S1

Text S1 - Modelling Equations

The full collection of the mathematical equations used for modelling the polarization response are provided below:

$$\mathbf{a} = \begin{pmatrix} cos(\phi) & sin(\phi) & 0 \\ -sin(\phi) & cos(\phi) & 0 \\ 0 & 0 & 1 \end{pmatrix} \begin{pmatrix} cos(\theta) & 0 & sin(\theta) \\ 0 & 1 & 0 \\ -sin(\theta) & 0 & cos(\theta) \end{pmatrix} \begin{pmatrix} cos(\psi) & sin(\psi) & 0 \\ -sin(\psi) & cos(\psi) & 0 \\ 0 & 0 & 1 \end{pmatrix}$$
$$= \begin{pmatrix} \cos(\theta)\cos(\phi)\cos(\psi) - \sin(\phi)\sin(\psi) & \cos(\theta)\cos(\psi)\sin(\phi) + \cos(\phi)\sin(\psi) & \cos(\psi)\sin(\theta) \\ -\cos(\psi)\sin(\phi) - \cos(\theta)\cos(\phi)\sin(\psi) & \cos(\phi)\cos(\psi) - \cos(\theta)\sin(\phi)\sin(\psi) & -\sin(\theta)\sin(\psi) \\ -\cos(\phi)\sin(\theta) & -\sin(\theta)\sin(\phi) & \cos(\theta) \end{pmatrix} \tag{S1}$$

$$\begin{pmatrix} E_x \\ E_y \\ E_z \end{pmatrix} = \begin{pmatrix} \cos(\theta)\cos(\phi)\cos(\psi) - \sin(\phi)\sin(\psi) & \cos(\theta)\cos(\psi)\sin(\phi) + \cos(\phi)\sin(\psi) \\ -\cos(\psi)\sin(\phi) - \cos(\theta)\cos(\phi)\sin(\psi) & \cos(\phi)\cos(\psi) - \cos(\theta)\sin(\phi)\sin(\psi) \\ -\cos(\phi)\sin(\theta) & -\sin(\theta)\sin(\phi) \end{pmatrix} \begin{pmatrix} E_X \\ E_Y \end{pmatrix} \tag{S2}$$

$$\begin{pmatrix} E_X \\ E_Y \end{pmatrix} = \begin{pmatrix} \cos\alpha \\ \sin\alpha \end{pmatrix} \tag{S3}$$

$$\chi^{(2)}_{ijk} = \begin{pmatrix} xxx & xyy & xzz & xyz & xxz & xxy \\ yxx & yyy & yzz & yyz & yxz & yxy \\ zxx & zyy & zzz & zyz & zxz & zxy \end{pmatrix} \tag{S4}$$

$$\chi^{(2)}_{ijk} = \begin{pmatrix} xxx & 0 & 0 & 0 & 0 & 0 \\ 0 & 0 & 0 & 0 & 0 & 0 \\ 0 & 0 & 0 & 0 & 0 & 0 \end{pmatrix} \tag{S5}$$

$$\chi^{(2)}_{ijk} = \begin{pmatrix} xxx & xyy & xzz & yzx & 0 & 0 \\ 0 & 0 & 0 & 0 & yzx & xyy \\ 0 & 0 & 0 & 0 & xzz & yzx \end{pmatrix} = \begin{pmatrix} xxx & Nxxx & Mxxx & Cxxx & 0 & 0 \\ 0 & 0 & 0 & 0 & Cxxx & Nxxx \\ 0 & 0 & 0 & 0 & Mxxx & Cxxx \end{pmatrix} \tag{S6}$$

$$\begin{pmatrix} P^{(2)}_x \\ P^{(2)}_y \\ P^{(2)}_z \end{pmatrix} = \chi^{(2)}_{ijk} \begin{pmatrix} E_x^2 \\ E_y^2 \\ E_z^2 \\ 2E_yE_z \\ 2E_xE_z \\ 2E_xE_y \end{pmatrix} \tag{S7}$$

$$\begin{pmatrix} P^{(2)}_X \\ P^{(2)}_Y \\ P^{(2)}_Z \end{pmatrix} = a^{-1} \begin{pmatrix} P^{(2)}_x \\ P^{(2)}_y \\ P^{(2)}_z \end{pmatrix} \tag{S8}$$

$$\begin{pmatrix} I_X \\ I_Y \end{pmatrix} \sim \begin{pmatrix} P^{(2)2}_X \\ P^{(2)2}_Y \end{pmatrix} \tag{S9}$$

- Equation S1 ZYZ Euler angle transformation matrix with ϕ rotation about the z-axis, θ rotation around the y axis and ψ rotation around the z axis. xyz correspond to the molecular frame coordinates.
- Equation S2 Calculating the microscopic electrical field components from the transformation matrix and the macroscopic filed components and assuming $E_Z = 0$. XYZ correspond to the macroscopic frame coordinates.

- Equation S3 The linear polarization of the macroscopic field components are expressed with angle α
- Equation S4 The second order susceptibility tensor, χ^2_{ijk}, in compressed form for the special case of the degenerated case of SHG whereby the two excitation fields are indiscernible ($\chi^2_{ijk} = \chi^2_{jik}$). Kleinmann symmetry holds true when all optical fields are far removed from any transition ($\chi^2_{kji} = \chi^2_{ijk}$).
- Equation S5 The second order susceptibility response of C_2 with xxx dominant
- Equation S6 The second order susceptibility response of C_2 with four independent tensor components, xxx, xyy, xzzz and yzx and also expressed with fit paramters M,N and C.
- Equation S7 The microscopic second order polarization response calculated from the microscopic field components and the second order susceptibility tensor
- Equation S8 The macroscopic second order polarization response from the transformation matrix of the microscopic second order polarization response
- Equation S9 The measurable intensity related to the square of the macroscopic polarization

Text S2 - Polarized Optical Micrsoscopy vs SHG Microscopy

The validation of SHG microscopy was conducted via comparative analysis with polarized optical microscopy (POM), an established technique widely used in similar studies. The microscope used was an Olympus BX-51 with an Olympus 20x/0.40 objective. The original POM images were cropped to coincide with the dimension of the equivalent SHG image. In the presented images, the line scans obtained from both SHG microscopy and POM revealed similar values for the periodicity of the structural features under investigation.

The values for the 8 wt.% lab preparation samples and the Celluforce 100L 4 wt.%, 3 wt.% and 2 wt.% samples are provided in Fig. S1. For the 8 wt.% lab prepared sample and the Celluforce 4 wt.% and 3 wt.%, only minor differences are observed. These minor difference arise from variations in the exact location being observed between SHG and POM. The samples were also measured on consecutive days, yet they had already settled for a week beforehand. Hence, it is unlikely that any further changes in periodicity would occur following such a settled period. Furthermore, the alignment is typically observed almost immediately after sample preparation, suggesting that a week's duration is more than enough to achieve equilibrium.

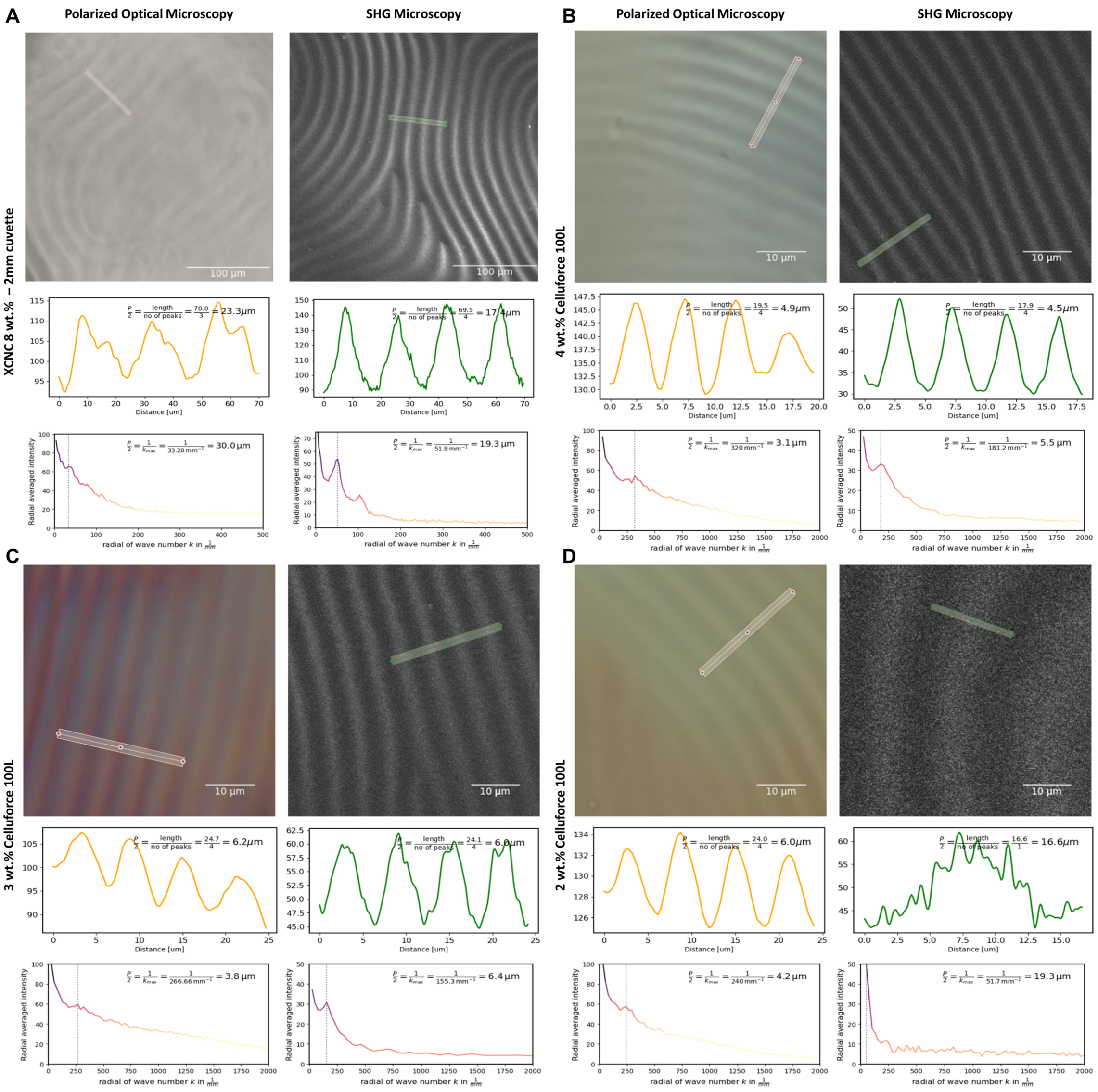

Figure S1. **Four quadrants featuring POM images, 2D SHG images, and their corresponding line scans and 2D-DFTs.** The quadrants correspond to chiral nematic CNC suspension in: **A** 8 wt.% lab-prepared in a quartz cuvette, **B** 4 wt.% Celluforce, **C** 3 wt.% Celluforce , and **D** 2 wt.% Celluforce

On the other hand we observed strong differences for the 2 wt.% Celluforce 100L. The explanation for this is in line with the conclusions of the main text. What we are observing in the SHG image are highly tilted chiral nematic domains. Indeed when looking at other regions of the 2 wt.% we see a lot of regions with no alignment. The chiral axis extends to a lesser extent than in the other samples and this would suggest that the domains are tilting i.e they are not consistenly laying perpendicular to the optical axis. Regarding the POM image, the chiral axis is more perpendicular to the optical axis than the SHG image, resulting in shorter pitch values being observed.

As seen in Fig. S1, identifying the maximum frequency component in the DFT of the POM is more challenging than identifying the sharply defined peak observed in the DFT of the SHG image. This is because the SHG image demonstrates enhanced contrast compared to POM images, resulting in more accuracte DFT analysis. This heightened contrast in the SHG image arises from the intrinsic confocal effect and the robust $\chi^{(2)}$ response exhibited by the aligned CNC rod. Additionally, the SHG image displays uniform brightness levels between each bright and dark line, further aiding in the analysis. Conversely, POM images lack such uniform brightness levels due to out-of-focus planes and the birefringence of the structure. The consistent brightness levels observed in the SHG image facilitate precise determination of spatial frequency components, ensuring accurate p/2 characterization by DFT.

Text S3 - Uniform pitch maintained in four directions

In Fig. S2, it is verified that the chiral axis is perpendicular to the optical axis. This is done by measuring the pitch values in all directions, as any tilt would manifest as difference in the p/2 values. This analysis was conducted across all regions to ensure that their alignment was consistent. For the four ROIs we get p/2 = 2.14, 2.15, 2.17 and 2.16 μm where the difference between the largest and smallest value is less than 1.38%. These findings allow us to prove that the helical axis (**m**) is within the plan i.e. $\theta = 90°$. These conditions are a prerequisiste for performing the polarimetry fitting using our equations outlined in text S8.

It is worth mentioning that this assessment could have alternatively been conducted through z-scanning above and below the focal plane of interest, but this particular test was not executed. However, confirming the accurate orientation of the domain in this manner is considered robust and reliable.

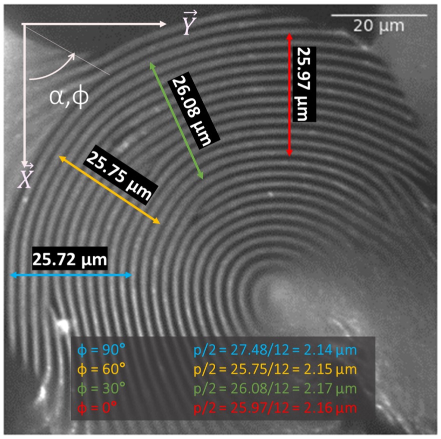

Figure S2. Pitch (p/2) is uniform across all orientations. The difference in each direction is less than 1.38%. This confirms that the helical axis, (m), is within the plane i.e. $\theta = 90°$.

Text S4 - Background correction

Studies found in literature utilizing SHG polarimetry typically involve examining crystals lying on a slide, with areas of empty space available for obtaining a background signal. This background signal is crucial for calibrating the setup, as it allows to determine whether the crystal response approaches zero, a factor directly linked to the components of its $\chi^{(2)}$ tensor. However in the case of a completely filled container, which is the case here in our CNC suspension, finding a blank is not as straight forward. Using a similar container filled with DI water is also not an option since the turbidity of the CNC suspension removes part of the light after the generation of the second harmonic light, and this also needs to be considered. In our ROIs of interest, a small region in the same focal plane was observed which did not show any alignment. It also showed minimal polarization repsonse. We have used this region of interest as our best option for probing a background in our system.

This area of interest is labeled as 'blank' and is depicted as a small purple circle in Fig.S3A with a zoomed-in view provided in Fig.S3B. This region exhibits minimal dependence on polarization. The polarization response is illustrated in Fig.S3C, where the standard deviation is plotted for each point alongside the average intensity. The average intensity at each polarization step serves as the primary plot. While minor intensity fluctuations are obesrved as a function of polarization, these differences are much samaller than the other ROIs. Specifically, the average intensity of the entire blank response is 27.5 [a.u] of I_{SHG}, with a maximum of 28.9 [a.u] and a minimum of 26.25 [a.u], which falls within the typical standard deviation, which is around 3 [a.u].

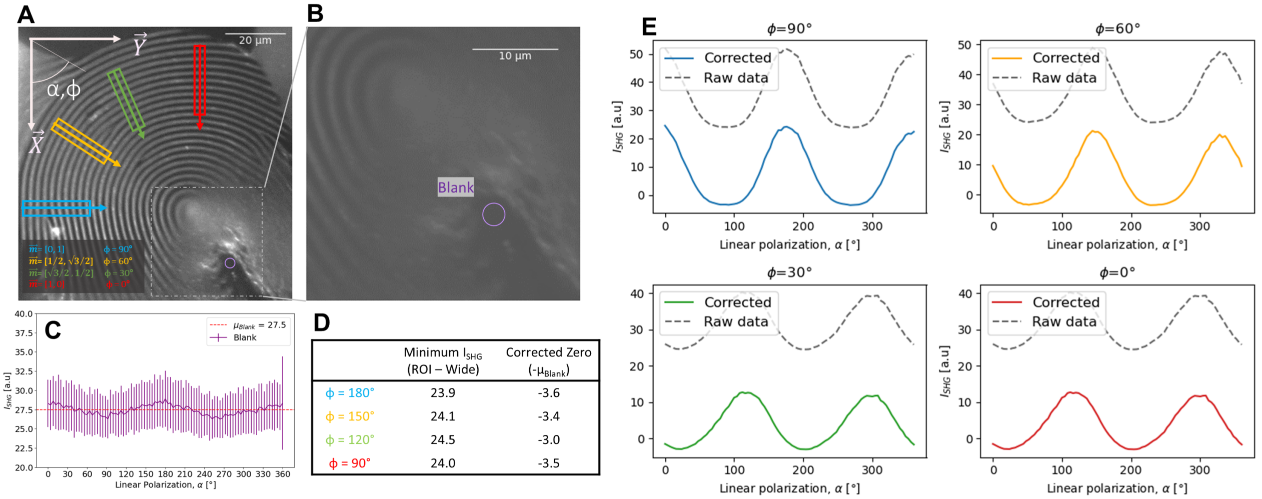

	Minimum I_{SHG} (ROI – Wide)	Corrected Zero ($-\mu_{Blank}$)
ϕ = 180°	23.9	-3.6
ϕ = 150°	24.1	-3.4
ϕ = 120°	24.5	-3.0
ϕ = 90°	24.0	-3.5

Figure S3. **Minima approaching zero aligns with dominant xxx response**. **A** The four ROIs positions. **B** Position of blank used is a small circular ROI with no visible alignment and low I_{SHG} signal **C** Polarization response within blank is minimal with an average value of μ_{Blank} = 27.5 [a.u] **D** Minimum points corrected by subtracting by μ_{Blank} for all plots. **E** Overview of polarization responses corrected to zero. It can be seen that the polarization responses approaches zero however with the blank correction the minima is located below zero. This is expected as the ROI used for the blank will contain some CNC rods in isotropic condition so the total I_{SHG} will not accurately describe the a pure water background solution.

Text S5 - Simulations: Polarization responses for a single dominant tensor response (xxx)

Solving Equation S9 with a single xxx dominant $\chi^{(2)}$ tensor component and θ =90° :

$$I_{SHG} = Axxx^2 sin^6(\psi) sin^4(\alpha - \phi) + E \tag{S10}$$

$$I_{tot} = Axxx^2 sin^4(\alpha - \phi) \int_{-\pi}^{\pi} sin^6(\psi) d\psi = Axxx^2 \frac{5\pi}{8} sin^4(\alpha - \phi) \tag{S11}$$

- Equation S10 simulates the total intensity response for in plane directors $\psi \neq 0$.
- Equation S11 simulates the total intensity response corresponding to all ψ orientations calculated as an integral of $-\pi$ to π.

The shape of the polarization response remains the same for all values $\psi \neq 0$ (eqn. S10) as well as the combined sum of all ψ orientations (eqn. S11). ϕ is expressed as a phase shift in the polarization response. The simulated curve correspond to the response measured in the CNC system.

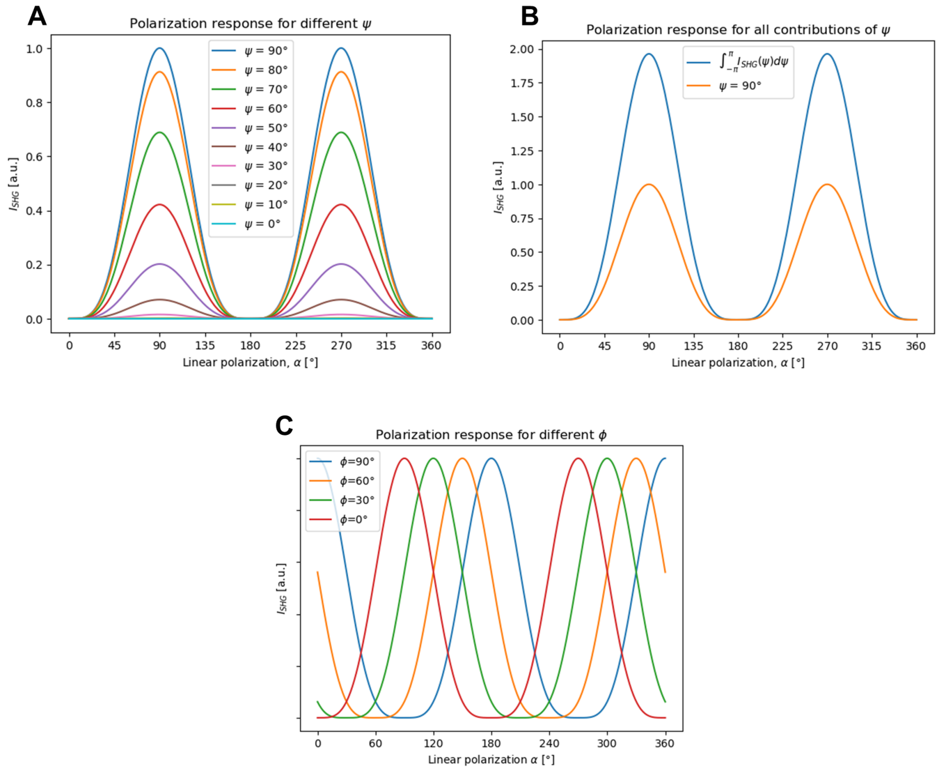

Figure S4. Simulation for dominant xxx. **A** Intensity response increases with anglee ψ. **B** Taking the contribution of all orientations of the rods provides equivalent observable response. **C** The aϕ translates to a phase shift.

Text S6 Simulations: In plane director (ψ) responses with M = xzz/xxx

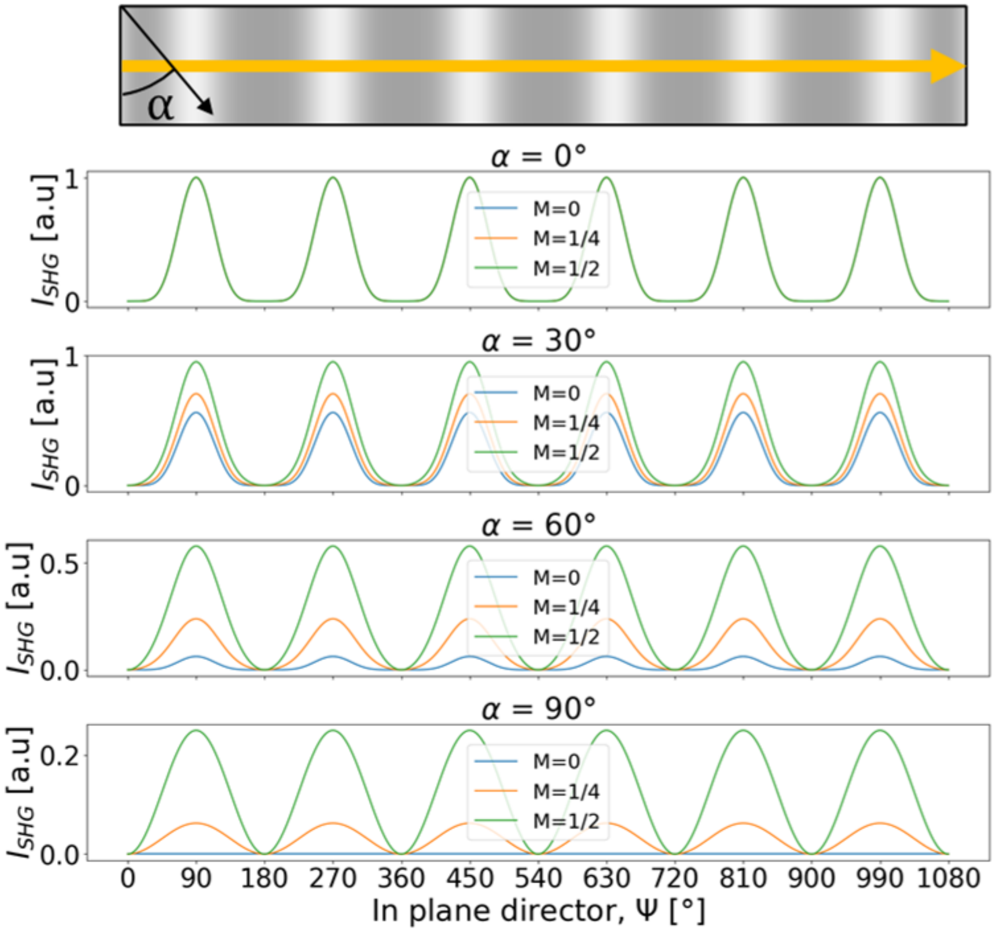

Figure S5. Simulation for M=xzz/xxx. In-plane director response for M = 0, 1/4 and 1/2. The distance between peaks remain constant across all polarization angles

The simulation curve for M indicates that when α is perpendicular to the chiral axis, the xzz/xxx off-tensor component does not affect the shape or intensity of the pattern. However, as the polarization aligns more with the chiral axis, we see both a change in intensity and a broadening of the profile, deviating from the typical sin^6 dependence. This broadening leads to sharper dips around the minima rather than the flatter profile seen initially. As the polarization changes, the profile shape varies, unlike when M=0, where the sin^6 function remains consistent across different polarizations. Importantly, there is no shift in the position or number of maxima and minima, meaning the profile still allows for accurate measurement of the system's pitch.

Text S6 Simulations: In plane director (ψ) responses with N = xyy/xxx

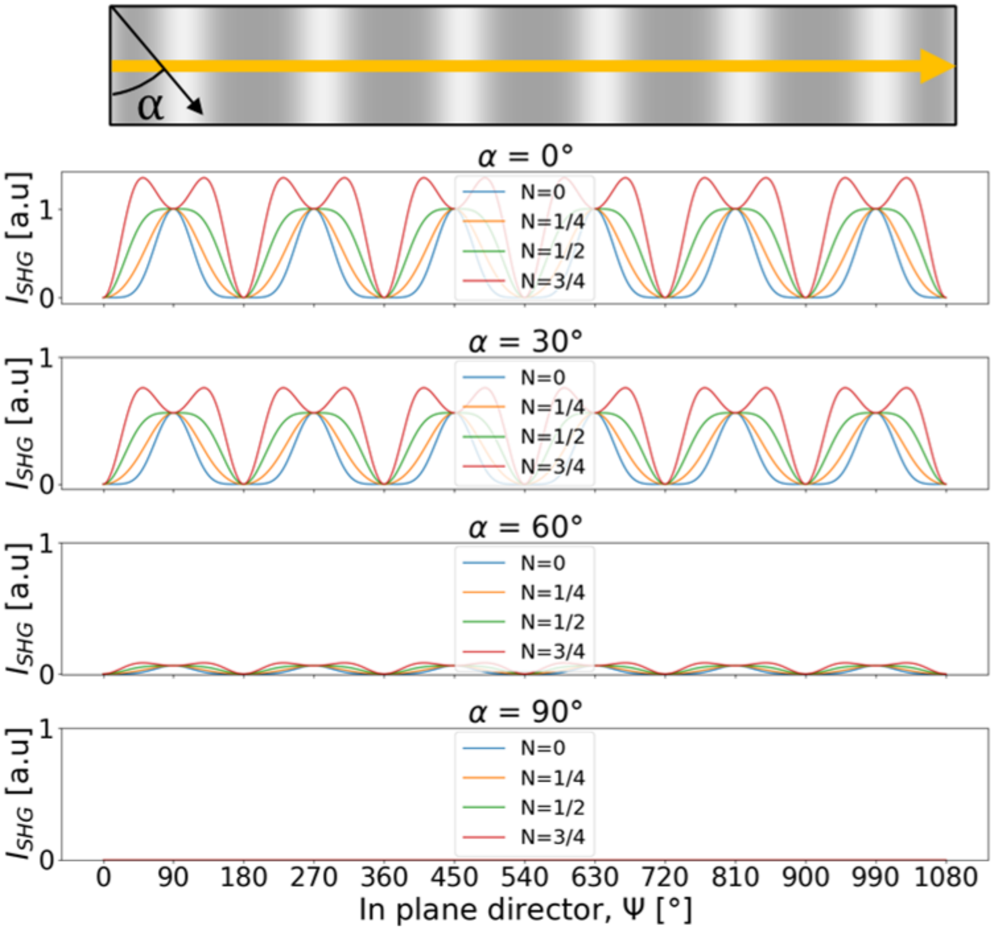

Figure S6. Simulation for N=xyy/xxx. In-plane director response for N = 0, 1/4 and 1/2. The distance between peaks remain constant across all polarization angles

The simulation curves for N shows that, unlike for M, deviations already occur at $\alpha = 0$. The limiting case is for N=1/2 which marks the occurrence of new maxima appearing in the profile. For N<1/2, the number and position of the maxima remains the same as in N=0. However for N>1/2, additional maxima within the profile start to emerge. These additional maxima do not align with the actual twisting of the rods, making it problematic for using the SHG images to characterize the system's pitch – so this scenario needs to be avoided (see). Unlike M where the shape of profile changes with varying α, here the profiles remains consistent across all polarization angles. Only instensity is reduced with varying α.

Text S6 Simulations: In plane director (ψ) responses with C = xyz/xxx

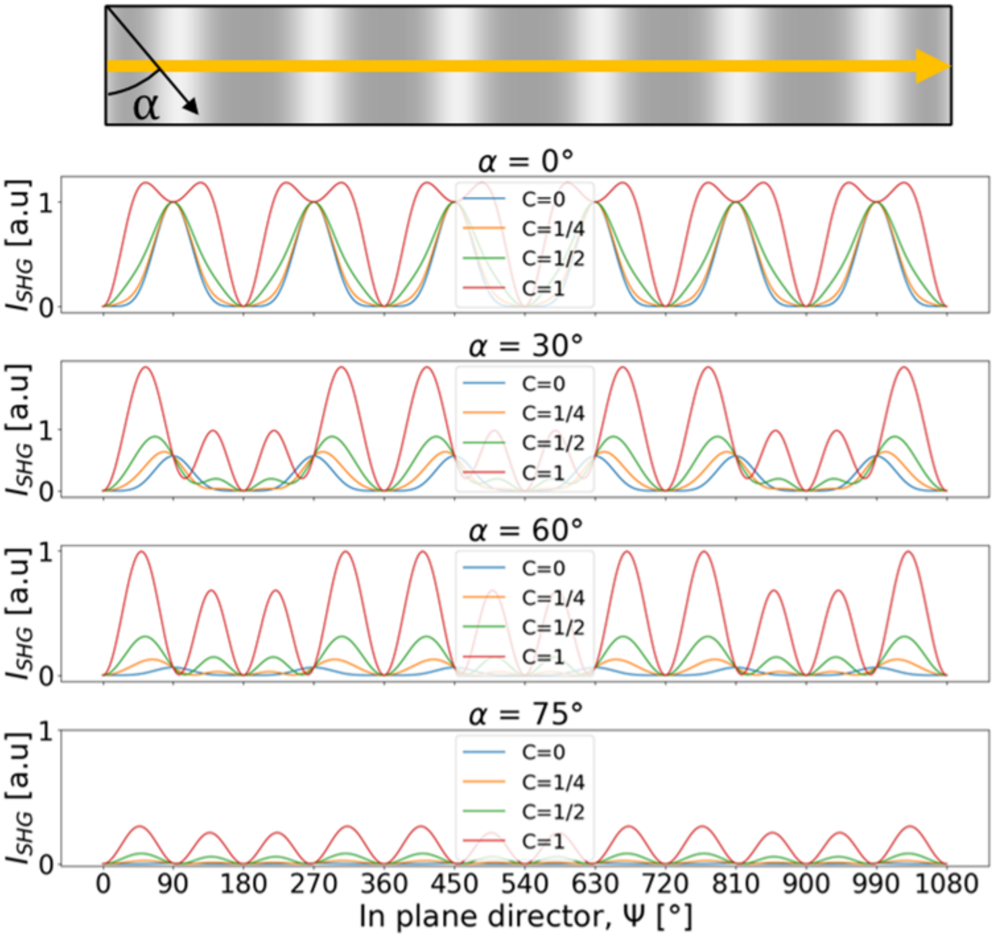

Figure S7. Simulation for C=xyz/xxx. In-plane director response for C = 0, 1/20 and 1/10. Even at small values of C, the distance between peaks start to shift. The distance between peaks will tend to double for α = 90° albeit with weaker signal.

The simulation curves for C reveal that the chiral components not only affect the shape and number of maxima but also significantly alter the maxima positions. A strong chiral contribution leads to additional peaks in the profile and shifts the location of all maxima, including those that are in line with the true physical orientation of the rods. Theoretically, with a sufficiently pronounced chiral effect, an SHG image could display distinct bands that are in reality half the size of the system's actual pitch. It is also interesting to see that the maxima positions move with varying alpha. This property will be used to show that such effects do not occur in our current CNC system.

Text S7 - Neglecting C : Uniform distance between peaks with polarization

We assume that the helical axis lies in the focal plane, as discussed earlier. First we aim to single out the influence of potential chiral components (i.e parameter C). These components have a distinct influence on the measured line profiles . Specifically, even small contributions of a component will tend to break the periodicity of the observed line patterns. Furthermore, the distance between the observed peaks in the line pattern will in this case depend on the polarization angle α. It is important to note that this effect is distinguished from the effect of off-axis components xzz and xyy, since these components preserve the periodicity of the line patterns, regardless of polarization angle (as simulated in text S6). We analyzed the pitch across all polarization angles and showed that for the chiral nematic CNCs no deviations in periodicity are apparent as illustrated in Fig. S8. This leads us to the conclusion that the contribution of the chiral xyz component is negligible (i.e C=0).

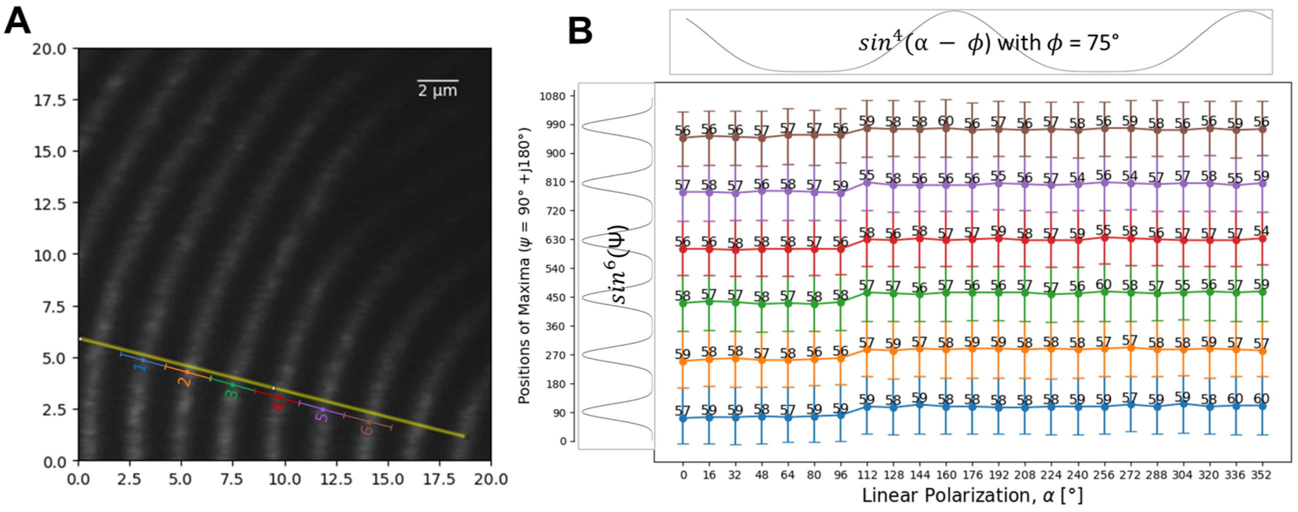

Figure S8. Tracking the distance between peaks. **A** Position of line scan with 6 peaks identified and labelled 1-6. **B** Distance between each peaks is expressed as pixel length. It was found that the distance between peaks remain constant over all polarization angles. For this reason, the chiral contribution from $C = xyz/xxx$ is negligible.

Text S8 - General solutions for C_2 with M and N fitting equations

$$
\begin{aligned}
I_{\mathrm{SHG}}(\psi,\phi,\alpha,xxx,N,M)\Big|_{\theta=90^\circ} = \; & xxx^2\sin^2(\psi) \\
& \times\Big[\Big(N\cos^2(\alpha-\phi)\cos(\phi)+\frac{1}{2}\cos(\phi)\left(1+3M+(-1+3M)\cos(2\psi)\right)\sin^2(\alpha-\phi) \\
& +N\sin(2(\alpha-\phi))\sin(\phi)\Big)^2+\big(N\cos(\phi)\sin(2(\alpha-\phi)) \\
& -\sin(\phi)\left(N\cos^2(\alpha-\phi)+\sin^2(\alpha-\phi)\left(3M\cos^2(\psi)+\sin^2(\psi)\right)\right)\big)^2\Big]
\end{aligned}
\tag{S12}
$$

$$
I_{\mathrm{SHG}}(\phi,\alpha,M)\Big|_{\substack{\theta=90^\circ\\ \Psi=90^\circ\\ xxx=1}} = \frac{1}{8}\left(3+M(2+7M)+4(-1+M^2)\cos(2(\alpha-\phi))-(1+M)(-1+3M)\cos(4(\alpha-\phi))\right)
\tag{S13}
$$

$$
\begin{aligned}
I_{\mathrm{SHG}}(\psi,N)\Bigg|_{\substack{\theta=90^\circ\\ xxx=1\\ \phi=\alpha+90^\circ}} = \int_{-\pi}^{\pi} I_{SHG}(\alpha)d\alpha = \; & \frac{1}{32}\pi\big(9+9N(2+9N)+8M+24NM+56M^2+ \\
& 4(-1+3N)(3+9N+2M)\cos(2\psi) \\
& +3(1-3N)^2\cos(4\psi)\big)\sin^2(\psi)
\end{aligned}
\tag{S14}
$$

- Equation S12 is obtained by taking C=0 and $\theta = 90°$. By solving equation S7 we get a general formula for the polarization response.
- Equation S13 is a simplifaction of Equation S12 by considering the in-plane director $\psi = 90°$. This results in N becoming isolated from M. By fitting the polarization responses and taking xxx =1 and for a known ϕ, M can be determined.
- Equation S14 is obtained by taking the average polarization response of all α contributions therefore eliminating the polarization dependence. We determine N by fitting to the in plane director response and using the parameter obtained for M from S13. The in plane director response corresponds to a line scan in the image.

Text S9 - Aperture angles of CNC

From our measurements the polar axis of the CNCs on average aligns along the X-axis in the XY plane. If this alignment was perfect,only an XXX component would be observed (under assumption of a pure xxx molecular component), however in reality we see a non-negligible XYY and a larger XZZ component. This component must then follow from a distribution along the polar X axis, projecting xxx onto the Y and Z-axes. In order to follow our convention the projection can be done based on two angles. $\frac{\sigma_1}{2}$ describe the angle with the X-axis, $\frac{\sigma_2}{2}$ is the angle in between the Z axis and the projection of x in the YZ plane. The projection of xxx onto XXX, XZZ and XYY then follows:

$$\begin{aligned} RxX &= xxx\cos\left(\frac{\sigma_1}{2}\right) \\ RxY &= xxx\sin\left(\frac{\sigma_1}{2}\right)\cos\left(\frac{\sigma_2}{2}\right) \\ RxZ &= xxx\sin\left(\frac{\sigma_1}{2}\right)\sin\left(\frac{\sigma_2}{2}\right) \end{aligned} \tag{S15}$$

$$\begin{aligned} XXX_p &= RxX \cdot RxX \cdot RxX = \cos\left(\frac{\sigma_1}{2}\right)^3 \\ XYY_p &= RxX \cdot RxY \cdot RxY = \cos\left(\frac{\sigma_1}{2}\right)\sin\left(\frac{\sigma_1}{2}\right)^2\cos\left(\frac{\sigma_2}{2}\right)^2 \\ XZZ_p &= RxX \cdot RxZ \cdot RxZ = \cos\left(\frac{\sigma_1}{2}\right)\sin\left(\frac{\sigma_1}{2}\right)^2\sin\left(\frac{\sigma_2}{2}\right)^2 \end{aligned} \tag{S16}$$

Two Dirac Delta functions are used for the integration of the distribution, one for angle $\frac{\sigma_1}{2}$ and one for angle $\frac{\sigma_2}{2}$

$$\begin{aligned} XXX &= \int_0^{2\pi}\int_0^{\pi} XXX_p \cdot \delta\left(\frac{\sigma_1}{2}-\frac{\sigma_{1,0}}{2}\right)\cdot\delta\left(\frac{\sigma_2}{2}-\frac{\sigma_{2,0}}{2}\right)\cdot\sin\left(\frac{\sigma_1}{2}\right)d\sigma_1\,d\sigma_2 = \sin\left(\frac{\sigma_{1,0}}{2}\right)\cos\left(\frac{\sigma_{1,0}}{2}\right)^3 \\ XYY &= \int_0^{2\pi}\int_0^{\pi} XYY_p \cdot \delta\left(\frac{\sigma_1}{2}-\frac{\sigma_{1,0}}{2}\right)\cdot\delta\left(\frac{\sigma_2}{2}-\frac{\sigma_{2,0}}{2}\right)\sin\left(\frac{\sigma_1}{2}\right)d\sigma_1\,d\sigma_2 = \cos\left(\frac{\sigma_{1,0}}{2}\right)\sin\left(\frac{\sigma_{1,0}}{2}\right)^3\cos\left(\frac{\sigma_{2,0}}{2}\right)^2 \\ XZZ &= \int_0^{2\pi}\int_0^{\pi} XZZ_p \cdot \delta\left(\frac{\sigma_1}{2}-\frac{\sigma_{1,0}}{2}\right)\cdot\delta\left(\frac{\sigma_2}{2}-\frac{\sigma_{2,0}}{2}\right)\sin\left(\frac{\sigma_1}{2}\right)d\sigma_1\,d\sigma_2 = \cos\left(\frac{\sigma_{1,0}}{2}\right)\sin\left(\frac{\sigma_{1,0}}{2}\right)^3\sin\left(\frac{\sigma_{2,0}}{2}\right)^2 \end{aligned} \tag{S17}$$

The values for M and N can then be used to calculate the aperture angles as follows:

$$\begin{aligned} M &= \frac{XZZ}{XXX} = \sin\left(\frac{\sigma_{2,0}}{2}\right)^2\tan\left(\frac{\sigma_{1,0}}{2}\right)^2 \\ N &= \frac{XYY}{XXX} = \cos\left(\frac{\sigma_{2,0}}{2}\right)^2\tan\left(\frac{\sigma_{1,0}}{2}\right)^2 \\ \frac{M}{N} &= \frac{XZZ}{XYY} = \tan\left(\frac{\sigma_{2,0}}{2}\right)^2 \end{aligned} \tag{S18}$$

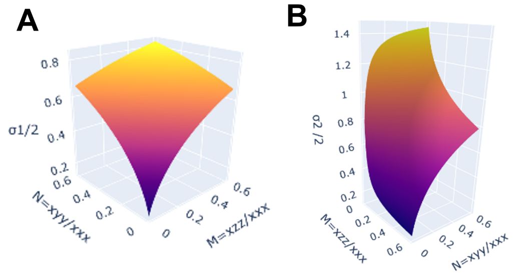

Figure S9. Solution space for aperture angles. Solutions for **A** $\frac{\sigma_{1,0}}{2}$ and **B** $\frac{\sigma_{2,0}}{2}$ with respect to M and N

Text S10 - Error propagation for aperture angles

The aperture angles $\frac{\sigma_{1,0}}{2}$ and $\frac{\sigma_{2,0}}{2}$ are calculated from eqn. S18. The errors for the fitting of M and N are neoted ΔM and ΔN are the values for the standard error obtained of the square root of the reduced Chi-Sqr of the non-linear polynomial fits. To obtain the error in the quotient for the formula $Q = M/N$:

$$\Delta Q = |Q|\sqrt{\left(\frac{\Delta M}{M}\right)^2 + \left(\frac{\Delta N}{N}\right)^2} \tag{S19}$$

When calculating the angle $\frac{\sigma_{2,0}}{2}$, the equation $f_2(Q) = arctan\left(\sqrt{Q}\right)$ is solved. The error $\Delta\frac{\sigma_{2,0}}{2}$ is calculated by the following:

$$\Delta\left(\frac{\sigma_{2,0}}{2}\right) = |\bar{f}_2'(Q)|\Delta Q = \frac{1}{2\sqrt{Q}(1+Q)}\Delta Q \tag{S20}$$

When calculating the angle $\frac{\sigma_{1,0}}{2}$, we solve the equation $f_1\left(M, \frac{\sigma_{2,0}}{2}\right) = arctan\left(\frac{\sqrt{M}}{sin\left(\frac{\sigma_{2,0}}{2}\right)}\right)$. The error $\Delta\frac{\sigma_{1,0}}{2}$ is then calculated by the following expansion:

$$\Delta\frac{\sigma_{1,0}}{2} = \frac{\partial f_1\left(M, \frac{\sigma_{2,0}}{2}\right)}{\partial M}\Delta M + \frac{\partial f_1\left(M, \frac{\sigma_{2,0}}{2}\right)}{\partial \frac{\sigma_{2,0}}{2}}\Delta\frac{\sigma_{2,0}}{2} \tag{S21}$$

Where the partial derivatives are expanded to the following:

$$\frac{\partial f_1}{\partial M} = \frac{1}{1+\left(\frac{\sqrt{M}}{\sin\left(\frac{\sigma_{2,0}}{2}\right)}\right)^2} \cdot \frac{1}{2\sqrt{M}} \cdot \frac{1}{\sin\left(\frac{\sigma_{2,0}}{2}\right)} \tag{S22}$$

$$\frac{\partial f_1}{\partial \frac{\sigma_{2,0}}{2}} = \frac{1}{1+\left(\frac{\sqrt{M}}{\sin\left(\frac{\sigma_{2,0}}{2}\right)}\right)^2} \cdot \left(-\frac{\sqrt{M}\cdot\cos\left(\frac{\sigma_{2,0}}{2}\right)}{\sin^2\left(\frac{\sigma_{2,0}}{2}\right)}\right) \tag{S23}$$

Considering all the above equations, the propagation of errors renders the following values:

ϕ	$M \pm \Delta M$	$N \pm \Delta N$	$Q \pm \Delta Q$	$\left(\frac{\sigma_{2,0}}{2}\right) + \Delta\left(\frac{\sigma_{2,0}}{2}\right)$	$\left(\frac{\sigma_{1,0}}{2}\right) + \Delta\left(\frac{\sigma_{1,0}}{2}\right)$
90°	0.079 ± 0.018	0.275 ± 0.018	0.29 ± 0.07	28.2 ± 2.83	30.8 ± 6.07
60°	0.061 ± 0.015	0.404 ± 0.013	0.15 ± 0.04	21.2 ± 2.40	34.3 ± 5.85
30°	0.209 ± 0.015	0.437 ± 0.022	0.48 ± 0.04	34.7 ± 1.17	38.8 ± 2.70
0°	0.179 ± 0.012	0.222 ± 0.024	0.81 ± 0.10	41.9 ± 1.81	32.3 ± 3.83

Table S1. Fit parameters and the calculated aperture angles with uncertainty

Text S11 - Apertures Angles of Celluforce

When comparing the aperture angles of 4 wt.% with those of the 3 wt.%, equivalent values of the $\frac{\sigma_{2,0}}{2}$ angles are measured but a significant increase in the $\frac{\sigma_{1,0}}{2}$. This could be expected since as the pitch decreases, the twisting angle between successive planes becomes tighter. Consequently, any natural deviations from perfect alignment are amplified, and the preferential alignment shifts more rapidly compared to the case of 3 wt.% .

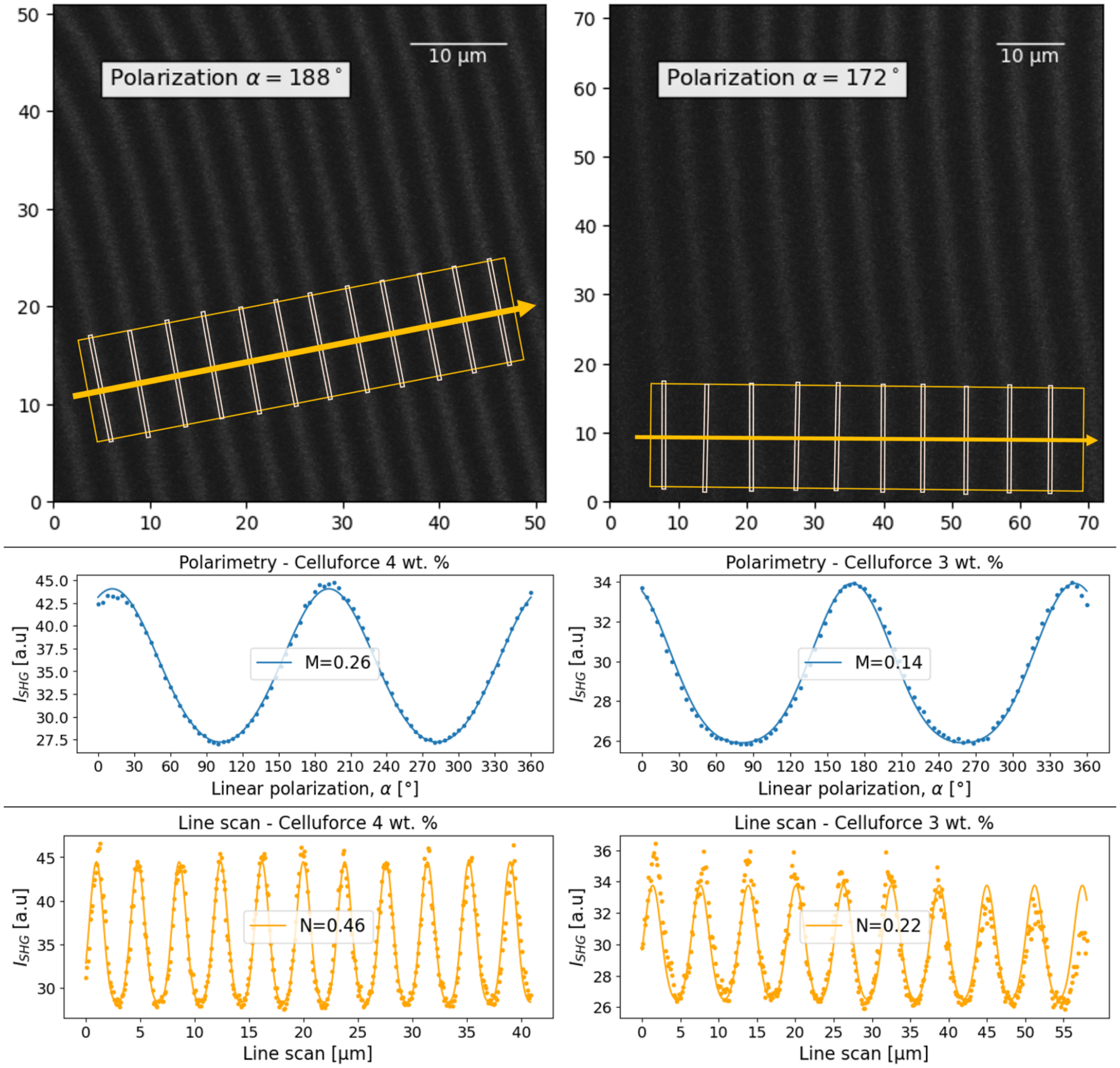

CelluForce 100L	M = xzz/xxx	N = xyy/xxx	$\sigma_{1,0}$ /2	$\sigma_{2,0}$ /2
4 wt. %	0.257 ± 0.007	0.458 ± 0.006	40.2 ± 0.98	36.9 ± 0.40
3 wt. %	0.144 ± 0.013	0.223 ± 0.017	31.2 ± 3.64	38.8 ± 1.69

Figure S10. Effect of aperture angle with concentration. Polarimetry was performed on Celluforce 4 wt.% (left) and 3 wt% (right) and the aperture angles were obtained for the fits of parameters M and N

Text S12 - Simulations : Line scans with linear and circular polarization

If we consider the polarization as circular we can express the electric field as the following:

$$\begin{pmatrix} E_X \\ E_Y \end{pmatrix} = \begin{pmatrix} -j + \cos\alpha \\ \sin\alpha \end{pmatrix} \tag{S24}$$

By solving equation S9 we can simulate the response for varying values of M and compare it with linear polarization.

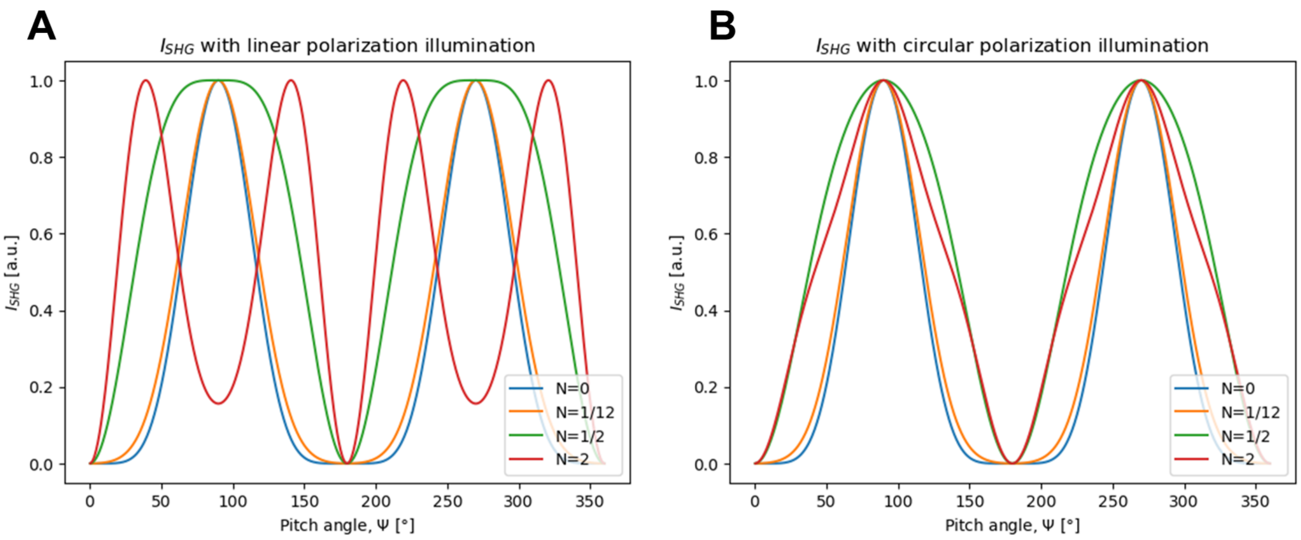

Figure S11. Simulations for Linear and Circular Polarization. A I_{SHG} as a function of pitch angle with linear polarized light **B** I_{SHG} as a function of pitch angle with circular polarized light. It is observed that the periodicity can always accurately be described for all values of M when circular polarization is used, however this is not true for high values of N (>1/2) when linear polarized light is used.

Simulations using circularly polarized light revealed an interesting finding: the pitch remains constant even at high values of N. This is not the case for linear polarization, where additional minima appear at values for N>0.5. Thus, the benefits for employing circularly polarized light for focus stacking in SHG microscopy are two fold. (1) It ensures uniform illumination of all orientations of the liquid crystal's chiral axis and (2) it maintains signal periodicity, even in highly disordered systems. In our previous analysis using linear polarization we did not encounter distortions of periodicity, since this limit was not reached (N<0.5). But this potential issue can be completely mitigated by using circular polarization.

Text S13 - Tomographic model demonstration

The 3D model generated by SHG microscopy can be loaded into tomography softwares to perform 3D analysis. Two free softwares were tried and these included the Zeiss Zen Blue 3.5 Edition and 3D Slicer 5.6.2.

Orthogonal slices of 8 wt.% lab prepared sample is shown with the Zen blue software in Fig.S12. In this model we can see the different orientations of the cut and this illustrates the benefits of tracking the defect in three dimensional space.

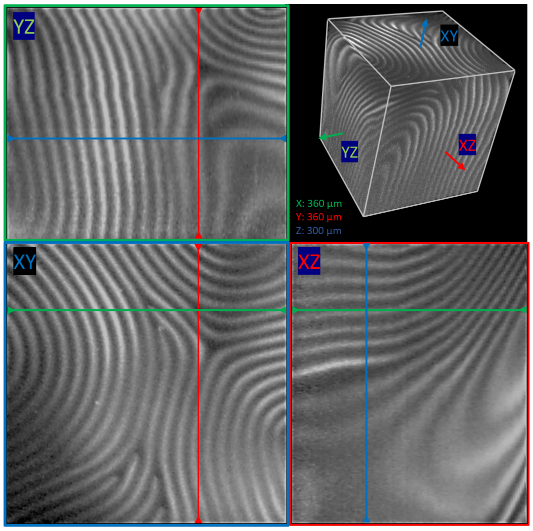

Figure S12. Tomographic orthogonal slicing. **A** Orthogonal slices YZ, XY and XZ of the model wt.% in the 2 mm quartz cuvette **B** Orthogonal slices YZ, XY and XZ of the model wt.% in the thin cuvette

Tilted projections were carried out with 3D Slicer 5.6.2 with the data obtained from the z-scanning of the Celluforce 3 wt.% sample. The measured value from the 3D-DFT generated a value of 5.2 µm ± 0.7 µm while the 2D-DFT in the optical plane generated a value of 6.4 µm ± 0.8 µm. By tilting the projections and aligning all orientations perpendicularly to the fringes, we can then do a line plot directly in one of the images. The line scan gives us a value of 49.66 µm across nine fringe. This gives us a value for p/2 = 5.5 µm which is consistent with the 3D measurement. Nonetheless, the 3D-DFT is the superior measuring technique since it considers the vairations of the pitch within the entire volume.

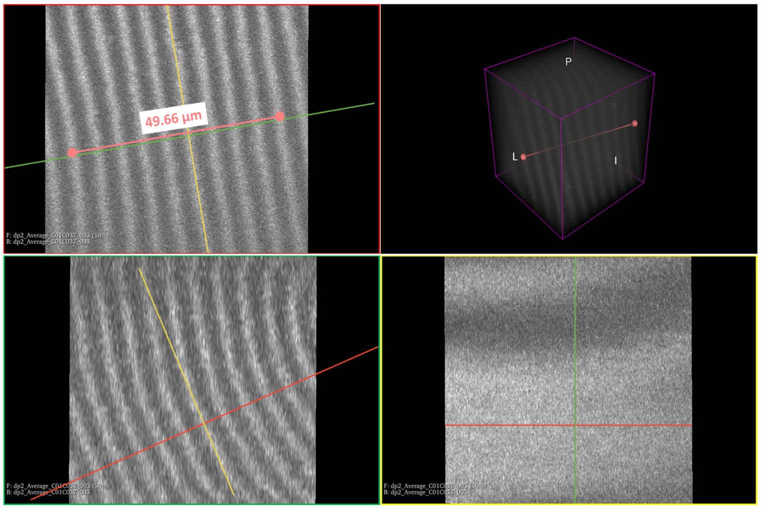

Figure S13. Tomographic tilted slicing. Tilting of domains to plot a line profile in 3D space for Celluforce 3 wt. %. The line scan can be seen at a tilted angle in the transparent 3D model.

Text S14 - Pitch distribution

The fitting was carried out by snipping at the edges of the peaks of the k-space radial profile and fitting to a gaussian and a second order polynomail. The equation to fit was the following:

$$y = C0 + C1x + C2x^2 + \frac{A}{w\sqrt{\frac{\pi}{2}}} \exp\left(-\frac{1}{2}\left(\frac{x - x_c}{w}\right)^2\right) \tag{S25}$$

A second-order polynomial was used to flatten the curve, and only the symmetric Gaussian component was retained for the k-space radial profile distribution. Taking the reciprocal to obtain the pitch distribution introduces skewness. Additionally, the distribution broadens for higher pitch dimensions. This is observed in Fig. S14. The reported standard deviation for pitch is defined as half of FWHM (Full Width at Half Maximum) of the skewed distribution. For instance, sample 4 wt. % has FWHM of 1.2 µm, therefore the reported variation for the pitch was reported as ± 0.6 µm.

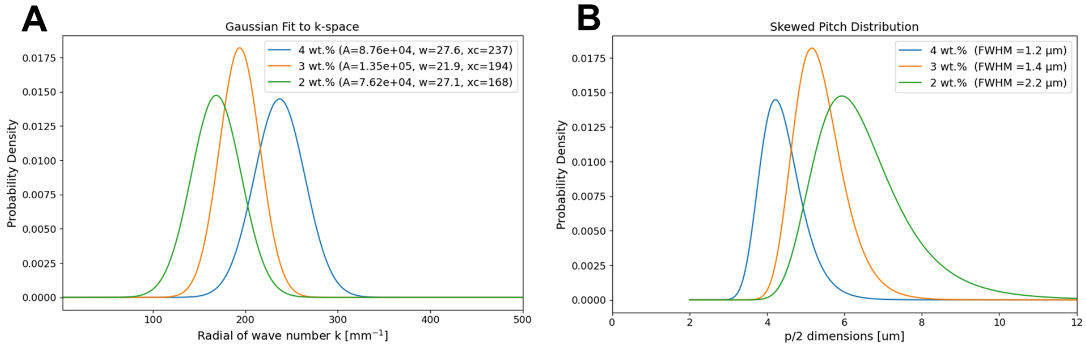

Figure S14. Pitch distribution. **A** Gaussian Distribution of the k-space **B** Skewed distribution of the pitch

Text S15 - Data Smoothening

Smoothing of the data is performed by weakening high frequencies in the spectral domain

$$F_{smooth}(\mathbf{k}) = F(\mathbf{k})exp(-k^2\, D) \tag{S26}$$

with the smoothing coefficient $D = 1 \times 10^{-4}\ mm^2$. We then perform the inverse DCT to obtain smoothed images. A smoothed slice of these data is provided in Fig. S16. The data smoothening was only carried out in the 8 wt.% sample in the cuvette and not in the CelluForce concentrataion series.

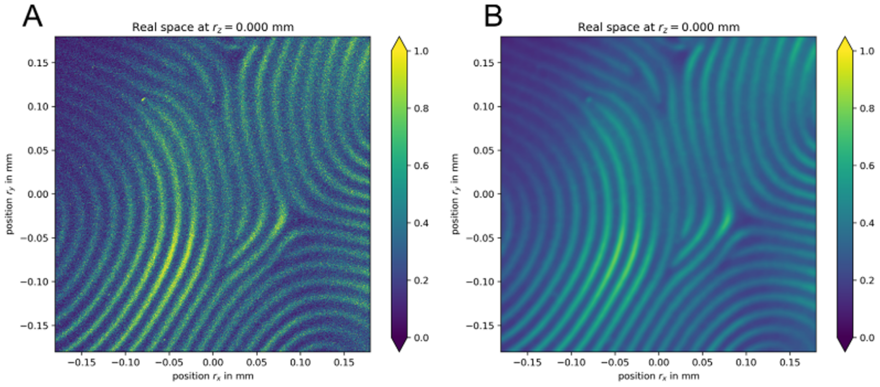

Figure S15. Data smoothening. XCNC in 2mm quartz cuvette **A** Raw image and **B** after data smoothening

Text S16 - Debye length and surface potential

- Number of sulfate groups per gram of CNC : $N_{SO_3-} = 0.158$ mmol/g (by conductometric titration)
- Zeta potential : ζ = -52 mV
- Denstiy of cyrstalline cellulose : 1.59 g/cm^3

If we consider 1 L of 8 wt.% CNC, we have 51.85 mL of CNC and a total weight of 82.4 g of CNC. The concentration of sulfates is then:

$$c_{So3-} = 0.158 \times \; 82.2 \;\; g \; = \; 13.0 \, mmol/L \tag{S27}$$

We have for an electrically neutral suspension:

$$c_{Na^+} = c_{SO_3^-} \tag{S28}$$

As an upper limit we consider all ions as free flowing and therefore contributiong to the ionic strength

$$I = \frac{1}{2}\sum c_i z_i^2 = 6.5 \times 10^{-3} \; mol/L \tag{S29}$$

The counter ions in suspension are attracted to the charged particles and a large number of these particles are fairly localized close to the particle. Entropic consideration however promotes the dissolution of the counter ions and an exponential decay is observed such that:

$$\phi\,(x) = \phi_0 exp\,(-\kappa x) \tag{S30}$$

The Debye screening length (κ^{-1}), which affects the electrostatic repulsion, is influenced by both the surface potential of the particles and the ionic strength of the solvent. It is expressed as the following:

$$\kappa^{-1} = \sqrt{\frac{\varepsilon_0 \varepsilon_r k_B T}{2q^2 I}} = \frac{0.304 \; nm}{\sqrt{I}} \, (in \; water \; at \; room \; T) \tag{S31}$$

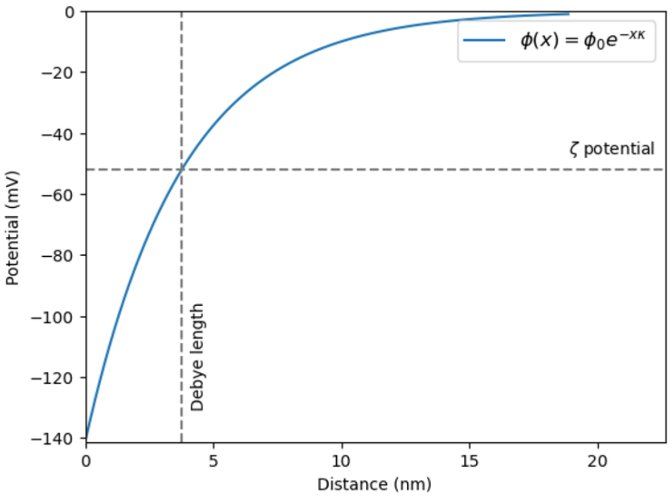

Figure S16. Potential close to CNC surface. The slipping plane is located after the Debye length, κ^{-1}.

$$\kappa^{-1} = \frac{0.304}{\sqrt{6.5 \times 10^{-3}}} \; = \; 3.77 nm \tag{S32}$$

$$\phi\left(\kappa^{-1}\right) = \phi_0 exp\left(-1\right) = 0.368 \times \phi_0 = \zeta = -52mV \rightarrow \phi_0 = -141\ mV \tag{S33}$$

The electric field can be expressed as:

$$E = -\frac{d\phi}{dx} = \kappa\phi_0 exp\left(-\kappa x\right) \tag{S34}$$

At the slipping plane (x= κ^{-1}), we get for the Electric field:

$$E_{DC} = \frac{-141\times10^{-3}}{3.77\times10^{-9}}\times0.368 = -13.76\times10^{6}\quad V/m \tag{S35}$$

Text S17 - Electric-Field Induced Second Harmonic from potential at the surface

It has been reported that ionic strength-dependent phase shifts in SHG can occur from charged interfaces whereby the dispersion of the fundamental and second harmonic beams modulates observed signals intensities. (*44,45*) The situation is similar to Ohno et al (*46*), describing interference between terms arising from a noncentrosymmetric bulk material (governed by $\chi^{(2)}_{\text{bulk}}$) and its interface (governed by $\chi^{(2)}_{\text{S}}$ and $\chi^{(3)}\Phi(0)$). From the work of de Coene et al (*42*) and Bloembergen (*47*), the non-resonant SHG from the bulk of the CNCs can be considered as a purely imaginary term, and we come to the following equation:

$$I_{SHG} \propto \left(\chi^{(2)}_{S}\right)^2 + \left(\chi^{(2)}_{\text{CNC,bulk}}\right)^2 \mp 2\phi_0\chi^{(2)}_{\text{CNC,bulk}}\chi^{(3)}_2 + 2\phi_0\chi^{(2)}_S\chi^{(3)}_1 + \phi_0^2\left(\chi^{(3)}_1\right)^2 + \phi_0^2\left(\chi^{(3)}_2\right)^2 \tag{S36}$$

The last two terms are negligible due to the squaring of $\chi^{(3)}$ terms, giving:

$$I_{SHG} \propto \left(\chi^{(2)}_{S}\right)^2 + \left(\chi^{(2)}_{\text{CNC,bulk}}\right)^2 \mp 2\phi_0\chi^{(2)}_{\text{CNC,bulk}}\chi^{(3)}_2 + 2\phi_0\chi^{(2)}_S\chi^{(3)}_1 \tag{S37}$$

The last two terms are described by the following equations:

$$\chi^{(3)}_1 = \frac{\kappa^2}{\kappa^2+(\Delta k_z)^2}\chi^{(3)} \tag{S38}$$

$$\chi^{(3)}_2 = \frac{\kappa\Delta k_z}{\kappa^2+(\Delta k_z)^2}\chi^{(3)} \tag{S39}$$

We see that the total SHG signal is proportional to the Debye screening length (κ^{-1}) and the wave vector mismatch (Δk_z). The phase mismatch between the fundamental and the second harmonic beams can be calculated by the k-vectors. If we consider index 1 to represent the harmonic and index 2 the fundamental, we express the phase mismatch as follows:

$$\Delta k = k_2 - 2k_1 = 2\left(\frac{2\pi n_1}{\lambda_1}\right) - \left(\frac{4\pi n_2}{\lambda_1}\right) = \frac{4\pi\left(n_1-n_2\right)}{\lambda_1} \tag{S40}$$

To calculate S40, we must consider the dispersion of water. We can use the well-known Sellmeir equation to obtain values for the refractive index at specific wavelengths:

$$n^2\left(\lambda\right) - 1 = \frac{B_1\lambda^2}{\lambda^2-C_1} + \frac{B_2\lambda^2}{\lambda^2-C_2} + \frac{B_3\lambda^2}{\lambda^2-C_3} \tag{S41}$$

With parameters (at room T): B1 = 0.56713427, B2 = 0.17716089, B3 = 0.00132137 & C1 = 0.0056983, C2 = 0.0173961, C3 = 160 (μm^2). For $\lambda_1 = 450$ nm and $\lambda_2 = 900$ nm, we get $n_1 = 1.3331782341683356$ and $n_2 = 1.323704948796715$. Which leads to the result:

$$\Delta k = \frac{4\pi\,(n1 - n2)}{\lambda_1} = 0.000264544\ (\text{nm}^{-1}) \tag{S42}$$

From our calculations for the Debye length of our CNCs , $\kappa^{-1} = 3.77$ (nm) (S32). By using these values and solving S38 and S39, we find:

$$\chi_1^{(3)} \approx 0.999999 \times \chi^{(3)}$$

$$\chi_2^{(3)} \approx 0.000997 \times \chi^{(3)}$$

Simplifying S37 further to obtain:

$$I_{SHG} \propto \left(\chi_S^{(2)}\right)^2 + \left(\chi_{\text{CNC,bulk}}^{(2)}\right)^2 + 2\chi_S^{(2)}\chi^{(3)}\Phi(0) \tag{S43}$$

This calculation corresponds to calculating the DC phase angle from the arctan of the wave vector mismatch by the length. This results in a very small angle (0.06°), as expected for negligible $\chi_2^{(3)}$.

According to a calculation of the different contributions by the group of Sylvie Roke (*48*), the first and the last term can be estimated from:

$$\chi_S^{(2)} \approx \chi^{(3)} \approx 10^{-18}\ (\text{esu})$$

$\chi_{\text{CNC,bulk}}^{(2)}$ can be estimated to be of the order of chiral biopolymers such as collagen, around 10^{-10} esu.(*49*) Hence it becomes clear that the second-order response of the bulk will overwhelmingly dominate that of the interface for the conditions used in our experiments.

One might argue that for certain polarizations the interfacial response might become non-negligible, as the response of the interface will become maximal when the polarization of the incident light is perpendicular to the longitudinal axis of the CNC (aligning with E_{DC}) , where the bulk response of the CNC becomes minimal (governed mainly by the xxx component along the longitudinal axis). However we would like to point out that the polarization patterns approach zero when the polarization is perpendicular to the longitudinal axis, as can be verified in Fig. 1 of the manuscript. This is an experimental indication that the response of the interface is negligible for the system under study.

Finally, based on the close proximity of the CNCs in the liquid crystalline mesophase, effective centrosymmetry is created with regards to the interfacial response, as the DC fields from the interface will point in opposite directions. For the interparticle distance in our system, this will result in a near complete cancellation of the second harmonic field. (*50*)
